# Acute Respiratory Infections Hospitalizations and In-Hospital Mortality in Ecuador: Differences in Patterns Between Pediatric and Adult Populations

**DOI:** 10.3390/medicina62091752

**Published:** 2026-09-11

**Authors:** German Josuet Lapo-Talledo, Andrés Ramírez, Jorge Andrés Talledo-Delgado, Karime Montes Escobar, Pedro Muñoz-Arteaga, Vanessa Quito-Calle, Lorena Cañizares-Jarrín, Andrea Margot Moreira Toscano

**Affiliations:** 1 Medicine Career, Pontificia Universidad Católica del Ecuador Sede Esmeraldas, Esmeraldas 080101, Ecuador; 2 Carrera de Psicología Clínica, Grupo de Investigación en Neurociencia Clínica Aplicada (GINCA), Universidad Politécnica Salesiana, Cuencua 010102, Ecuador; 3 School of Medicine, Faculty of Health Sciences, Universidad Técnica de Manabí, Portoviejo 130105, Ecuador; 4 Departamento de Formación y Desarrollo Científico en Ingeniería, Facultad de Ingeniería Ciencia y Tecnología, Universidad Bernardo O’Higgins, Santiago 8370993, Chile; 5 Laboratorio de Funcionamiento de Agroecosistemas y Cambio Climático-FAGROCLIM, Departamento de Ciencias Agrícolas, Facultad de Ingeniería Agrícola, Universidad Técnica de Manabí, Portoviejo 130105, Ecuador; 6 Facultad de Posgrado, Universidad Técnica de Manabí, Portoviejo 130105, Ecuador

**Keywords:** acute respiratory infections, respiratory tract infections, hospitalizations, in-hospital mortality, annual percentage change, Ecuador

## Abstract

*Background and Objectives*: Acute respiratory infections (ARIs) remain a leading cause of hospitalization and preventable in-hospital mortality. This study aimed to characterize ARI hospitalizations and mortality in Ecuador and to identify factors associated with mortality across age groups. *Materials and Methods*: A cross-sectional study was conducted using national hospital data for 2015–2023. Crude hospitalization and in-hospital mortality rates were estimated by year and province. Age-adjusted temporal trends were evaluated using Joinpoint regression to obtain annual percentage change (APC). Univariable and multivariable logistic regression analyses were used to assess factors associated with in-hospital mortality in the overall, pediatric and adult populations. *Results*: A total of 576,195 ARI hospitalizations were analyzed: 51.24% were pediatric and 48.76% adult admissions. Although children accounted for most hospitalizations, in-hospital mortality was substantially higher among adults (14.04%) than among children (0.53%) and increased progressively with age. Age-adjusted rates had a significant annual increase among adults from 2015 to 2021 (APC 30.08% for hospitalization and 55.10% for mortality) and declined significantly from 2021 to 2023 (APC: −53.09% and −72.90%, respectively), whereas pediatric trends showed no significant changes. Lower respiratory tract infections, including bacterial pneumonia, viral pneumonia, and COVID-19, showed the highest odds of in-hospital death. Bronchiolitis was associated with increased odds of death in both pediatric and adult populations, with markedly stronger associations among adults. Rural residence and ethnicity showed age-specific associations with mortality, potentially reflecting differences in access to hospital care. During 2020–2021, COVID-19 accounted for 70.71% of ARI hospitalizations and nearly 87.17% of in-hospital deaths, with ARI hospitalization rates remaining above pre-pandemic levels thereafter. *Conclusions*: ARIs impose a substantial and unequal burden on Ecuador’s health system. Mortality patterns may reflect age-related vulnerability, disease severity, and structural barriers to care rather than hospitalization volume alone, supporting the need for age-differentiated and equity-oriented public health strategies.

## 1. Introduction

Acute respiratory infections (ARIs) are among the leading causes of morbidity and mortality worldwide, disproportionately affecting the most vulnerable age groups, particularly children under five years of age and older adults [1]. According to estimates from the World Health Organization (WHO), ARIs account for more than 20% of all hospitalizations due to infectious diseases and nearly 15% of preventable deaths in low- and middle-income countries [2]. These conditions, which range from mild upper respiratory infections to severe pneumonia, are closely linked to social, environmental, and structural determinants that influence both their incidence and clinical outcomes, placing a substantial burden on health systems globally [3].

Over recent decades, the epidemiological dynamics of ARIs have undergone significant changes worldwide, driven by factors such as rapid urbanization, environmental pollution, population aging, and persistent inequalities in access to healthcare services [4]. Systematic reviews and meta-analyses have shown that, although the burden of disease remains high among pediatric populations [5], in-hospital mortality is disproportionately concentrated among adults and older individuals [6], in whom chronic comorbidities, such as diabetes [7], chronic obstructive pulmonary disease [8], and heart failure [9], substantially worsen the clinical course. This pattern highlights the importance of examining age-related differences in hospitalization patterns and clinical outcomes.

In the Latin American context, evidence derived from hospital-based ARI statistics has revealed marked heterogeneity in determinants and outcomes across regions, reflecting underlying sociodemographic disparities [10]. Studies conducted in countries such as Peru, Colombia, and Chile indicate that differences in hospital care, availability of intensive care units, and the quality of surveillance systems play a significant role in shaping in-hospital mortality [3,4,10]. However, in Ecuador, research addressing ARIs from a comparative age-group perspective remains limited and fragmented, constraining a comprehensive understanding of the differential factors influencing disease progression and mortality among hospitalized patients [11,12,13].

Analyzing ARI-related hospitalizations and in-hospital mortality in Ecuador is therefore of strategic importance for public health. Such analyses enable the identification of epidemiological patterns, the evaluation of clinical interventions, and more informed allocation of healthcare resources [14]. Differences between pediatric and adult populations may reflect not only age-related biological and clinical variability, but also inequities in timely access to hospital services and quality of care. Understanding these contrasts is essential for designing age-specific public policies that strengthen the national health system’s response to future respiratory disease outbreaks, particularly in the post-pandemic context characterized by emerging and persistent respiratory vulnerabilities [4]. The aim of this study was to conduct a sociodemographic analysis of hospitalizations due to acute respiratory infections and their associated in-hospital mortality in Ecuador, comparing epidemiological patterns between pediatric and adult populations. By using nationally available administrative databases from 2015 to 2023, this study provides an updated and comprehensive overview of the impact of acute respiratory infections on the Ecuadorian hospital system.

## 2. Materials and Methods

### 2.1. Study Design

This cross-sectional study analyzed registries of hospital discharges in Ecuador between 2015 and 2023. This study included individuals hospitalized due to a diagnosis of “Acute Respiratory Infections” according to the International Statistical Classification of Diseases 10th Revision (ICD-10) codes: J00-J06; J09-J18; J20-J22; J85-J86; U07.1-U07.2. Data were obtained from the Statistical Registry of Hospital Beds and Discharges of the Ecuadorian National Institute of Statistics and Censuses (INEC), which is available for public access (https://www.ecuadorencifras.gob.ec/camas-y-egresos-hospitalarios/) (accessed on 1 January 2025).

### 2.2. Study Sample

This study comprised hospitalization records for patients with a diagnosis of acute respiratory infection that were hospitalized in public and private healthcare centers across Ecuador between 2015 and 2023. A total of 576,195 hospitalizations due to acute respiratory infection were recorded between 2015 and 2023. The unit of analysis was the hospitalization records. The inclusion criteria were based on the predefined ICD-10 codes listed above, including U07.1-U07.2, which were introduced in 2020 for COVID-19 and remained the codes currently used to identify COVID-19 hospitalizations in the post-2020 INEC databases analyzed in this study. The ICD-10 codes considered for this study were J00-J06; J09-J18; J20-J22; J85-J86; and U07.1-U07.2. For subgroup analyses, pediatric patients were defined as those aged 0–19 years, whereas adults were defined as those aged ≥20 years.

### 2.3. Variables

The patient’s condition at the time of hospital discharge (alive or dead) was the dependent variable. Age groups, sex, ethnicity, area of residence (urban or rural), healthcare sector (public or private), type of acute respiratory infection, and year of hospital admission were independent variables. The variable “type of acute respiratory infection” was categorized into acute upper respiratory infections (ICD-10: J00-J06); influenza (ICD-10: J09-J11); viral pneumonia (ICD-10: J12); bacterial pneumonia (ICD-10: J13-J15); other pneumonias due to other or unspecified organisms (ICD-10: J16-J18); acute bronchitis (ICD-10: J20); acute bronchiolitis (ICD-10: J21); unspecified acute lower respiratory infection (ICD-10: J22); suppurative and necrotic conditions of lower respiratory tract (ICD-10: J85-J86); and COVID-19 (ICD-10: U07.1-U07.2).

### 2.4. Statistical Analysis

Continuous variable descriptive analyses were expressed as median and interquartile range (IQR), and categorical variables were given as frequency (n) and percentage (%). Categorical variables were compared using the Pearson χ^2^ test. Hospitalization rates and in-hospital mortality rates were calculated by province and calendar year using INEC’s 2024 revision of Ecuador’s population estimates and projections.

This review incorporates all available demographic information sources from 1950 to 2022, including the results of Ecuador’s VIII Population Census and VII Housing Census of 2022 [15].

Rates were calculated with the following formulas:

$$Hospitalization\;rate=\;\left(\frac{Number\;of\;cause\;specific\;hospitalizations}{Number\;of\;people\;in\;the\;population\;at\;risk}\right)\times 100,000$$

$$Inhospital\;mortality\;rate=\left(\frac{Number\;of\;cause\;specific\;inhospital\;deaths}{Number\;of\;people\;in\;the\;population\;at\;risk}\right)\times 100,000$$

In-hospital mortality proportions were calculated as the percentage of ARI-related hospitalizations resulting in death, whereas population based in-hospital mortality rates were expressed as deaths per 100,000 inhabitants.

Crude hospitalization rates and in-hospital mortality rates for pediatric and adult populations were calculated using the corresponding age-specific population projections from INEC. For the 2015–2023 period, the mean annual rate was calculated as the arithmetic mean of the nine annual crude rates. Additionally, age-adjusted ARI hospitalization rates and age-adjusted ARI in-hospital mortality rates for each calendar year were calculated using Joinpoint Trend analysis Software, version 6.1.0 [16]. The software was also used to assess temporal trends and perform Joinpoint regression, estimating annual percentage changes (APCs) and average annual percentage changes (AAPCs) with their corresponding 95% confidence intervals (95% CIs). For the calculation of age-adjusted rates, Ecuadorian population estimates by age group and calendar year were obtained from the population projections of the INEC [15]. The recommended World Health Organization (WHO) standard population for age-adjusted rates was obtained from the National Cancer Institute [17]. Model selection for the Joinpoint regression was based on the Weighted Bayesian Information Criterion (WBIC), which combines the conventional Bayesian Information Criterion (BIC) and BIC3 using a weighted penalty according to the characteristics of the data [18]. The model with the lowest WBIC was selected as the final Joinpoint regression model.

Univariable and multivariable binary logistic regression models were used to calculate the crude odds ratios (ORs) and adjusted odds ratios (aORs) for factors associated with acute respiratory infections in-hospital mortality (alive = 0 vs. dead = 1). Logistic regression models were performed as follows: one model for the whole study sample, one model for the pediatric sample and another model for the adult sample. Multicollinearity among independent variables was assessed using the Variance Inflation Factor (VIF) for each study sample. For the whole sample, the mean VIF was 2.31. The independent variables on the adult sample showed a mean VIF of 2.55, while on the pediatric sample there was a mean VIF of 1.41. The selection of independent variables entered into the multivariable binary logistic regression model was based on significant variables in the univariable logistic regression analyses with *p*-value < 0.20 and based on previous research on factors that influence acute respiratory infections adverse events. All *p*-values < 0.05 were considered statistically significant. The data were analyzed using the SPSS statistical program version 25.0 for Windows.

### 2.5. Ethical Considerations

This study did not require ethical approval according to local and international regulations. The data were obtained from publicly available secondary sources that do not contain sensitive or identifiable personal information. Research involving de-identified data is not classified as human subject research, thereby exempting it from ethical approval [19]. 

## 3. Results

### 3.1. Descriptive Data

Between 2015 and 2023, a total of 576,195 hospitalizations for acute respiratory infections were recorded in Ecuador, of which 41,048 (7.12%) resulted in in-hospital death. Of all admissions, 295,225 (51.24%) involved pediatric hospitalization records and 280,970 (48.76%) adult hospitalizations; the death proportion in the pediatric sample was markedly lower at 0.53% (n = 1575) versus 14.04% (n = 39,473) in adults. The overall median age was 14 years (IQR: 1–64); in pediatrics the median age was 1 year (IQR: 0–4) among survivors and 1 year (IQR: 0–5) among non-survivors, while in adults it was 63 years (IQR: 47–78) for survivors and 72 years (IQR: 61–83) for those who died. Age-group distributions also differed by population: 79.55% (n = 234,850) of pediatric admissions occurred in children aged 0–4 years, followed by 14.07% (n = 41,534) aged 5–9 years; among adults, 41.84% (n = 117,569) were ≥70 years, 17.54% were aged 60–69 years, 15.03% were aged 50–59 years and 10.92% were aged 40–49 years (Table 1).

Males comprised 53.73% (n = 309,593) of all hospitalizations and exhibited higher overall death proportions (7.91%) than females (6.21%); this sex difference was non-significant in pediatrics (males: 0.54% vs. females: 0.53%, *p* = 0.785) but remained significant in adults (males: 15.95% vs. females: 11.93%, *p* ≤ 0.001).

By ethnicity, Mestizos accounted for 79.46% of cases, Indigenous 3.88%, Black/Afro 0.51%, Montubio 0.27%, White 0.98%, other 3.83%, and unknown 11.07%. Overall, death proportions among ethnic groups were highest in other ethnicities that did not identify as one of the main ethnic groups described (8.81%), followed by Mestizos (7.25%). They were lowest in Indigenous (3.11%) and Montubio individuals (3.65%).

The most frequent respiratory infection subtype across the entire 2015–2023 period was “other pneumonias due to other or unspecified organisms” (40.68%), followed by COVID-19 (23.70%), bacterial pneumonia (12.47%), acute upper respiratory infections (7.48%), acute bronchitis (4.50%), unspecified lower respiratory infection (3.31%), acute bronchiolitis (3.05%), viral pneumonia (2.93%), influenza (1.50%), and suppurative/necrotic conditions (0.38%). In the pediatric population, “other pneumonias due to other or unspecified organisms” likewise predominated (49.04%), with bacterial pneumonia (13.48%), acute upper respiratory infections (11.42%), acute bronchitis (6.72%) and acute bronchiolitis (5.83%) completing the top five. Among adults, COVID-19 accounted for 46.06% of acute respiratory infections hospitalizations, followed by other pneumonias due to other or unspecified organisms (31.90%), bacterial pneumonia (11.40%) and acute upper respiratory infections (3.34%). Across the full 2015–2023 period, COVID-19 accounted for 23.70% of all hospitalizations. However, because there were no COVID-19 admissions before 2020, those four years of cases are spread across a nine-year window, diluting COVID-19′s apparent share. Consequently, when restricted to the 2020–2021 window, COVID-19 represented 70.71% of ARI hospitalizations and 87.17% of ARI in-hospital deaths. During 2020–2021, COVID-19 represented 14.26% of all pediatric acute respiratory infection admissions and 29.58% of all ARI pediatric deaths. For adults, during 2020–2021, COVID-19 represented 82.79% of all adult acute respiratory infection admissions and 87.93% of all ARI adult deaths. During this period of 2020–2021 pediatric COVID-19 death proportion was 2.46% and adult COVID-19 death proportion was 20.35%. During 2022–2023 COVID-19 represented 12.64% of ARI hospitalizations and 36.86% of ARI in-hospital deaths. During 2022–2023, COVID-19 represented 3.39% of all pediatric acute respiratory infection admissions and 7.16% of all ARI pediatric deaths. For adults, during 2022–2023, COVID-19 represented 29.58% of all adult acute respiratory infection admissions and 39.05% of all ARI adult deaths. During this period of 2022–2023 pediatric COVID-19 death proportion was 0.90% and adult COVID-19 death proportion was 13.54%.

### 3.2. Acute Respiratory Infections Hospitalizations and In-Hospital Mortality Rates

For the total population, the mean hospitalization rate between 2015 and 2023 for acute respiratory infections was 371.23 hospitalizations per 100,000 inhabitants. Provinces with the highest mean hospitalization rates were Morona Santiago (843.48), Cañar (599.62), Azuay (559.51), Tungurahua (529.16), and Pichincha (524.47), while the lowest were Esmeraldas (191.87), Orellana (197.15), Los Ríos (236.58), Santa Elena (244.11) and Manabí (254.78) (Table 2 and Supplementary Table S1). Annual rates increased sharply from 295.19 hospitalizations per 100,000 inhabitants in 2015 to 492.89 in 2020 and 473.70 in 2021, before subsequently falling to 361.42 in 2022 and 371.58 in 2023 after peaking during the COVID-19 surge. The mean in-hospital mortality rate was 26.18 per 100,000 inhabitants between 2015 and 2023, highest in El Oro (53.60), Tungurahua (48.18), Azuay (46.57), Cañar (35.30) and Chimborazo (34.32) and lowest in Orellana (9.34), Los Ríos (11.90), Esmeraldas (13.68), Bolívar (14.28) and Cotopaxi (14.31); mortality peaked in 2020 with 75.66 and 2021 78.92 in-hospital deaths per 100,000 inhabitants before declining in 2022 with 18.97 and 2023 with 9.58 in-hospital deaths (Table 2 and Supplementary Table S2).

**Figure 1:**
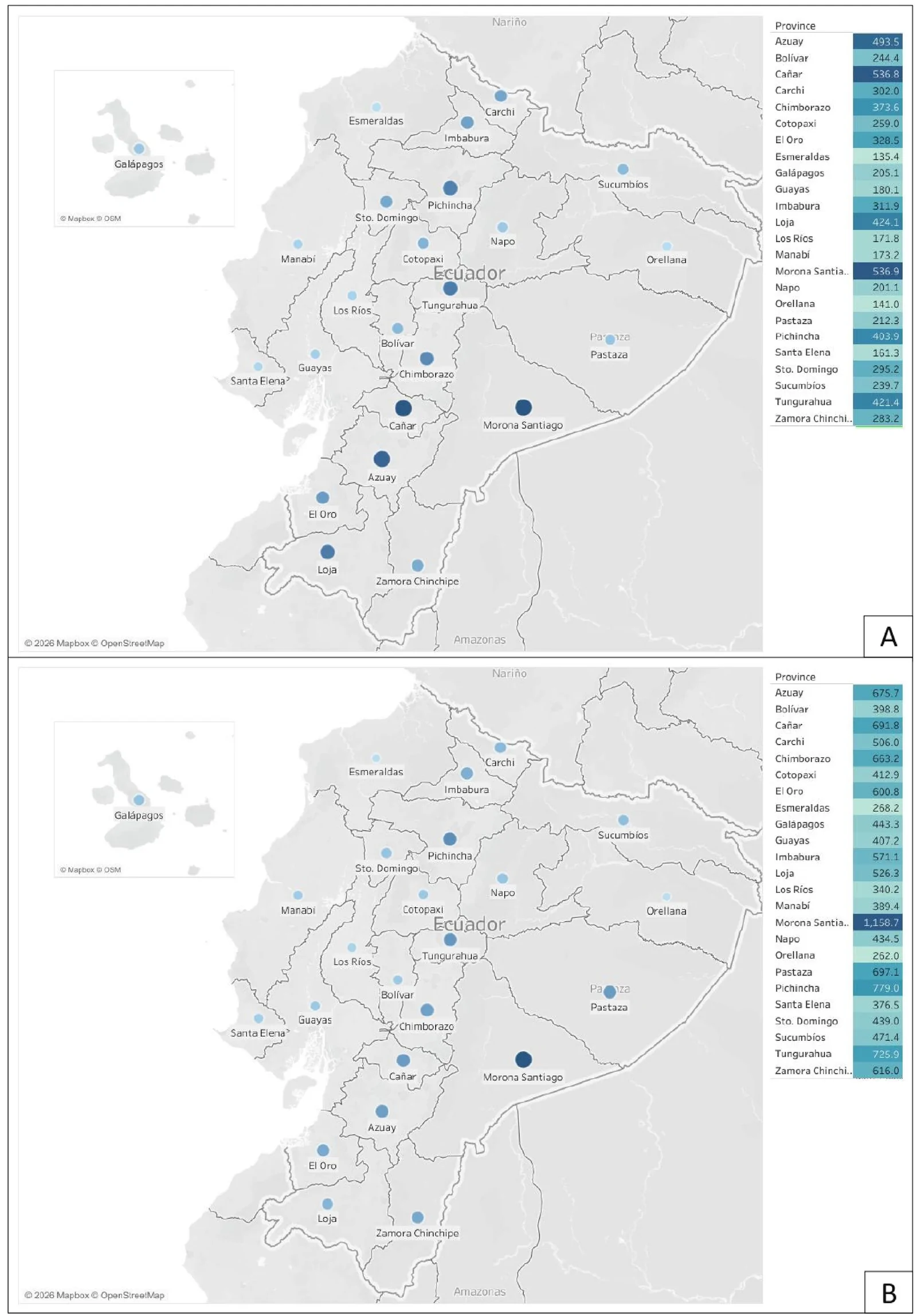
Mean crude acute respiratory infection (ARI) hospitalization rates per 100,000 population by province, Ecuador, 2015–2023: (**A**) adults and (**B**) pediatric population.

**Figure 2:**
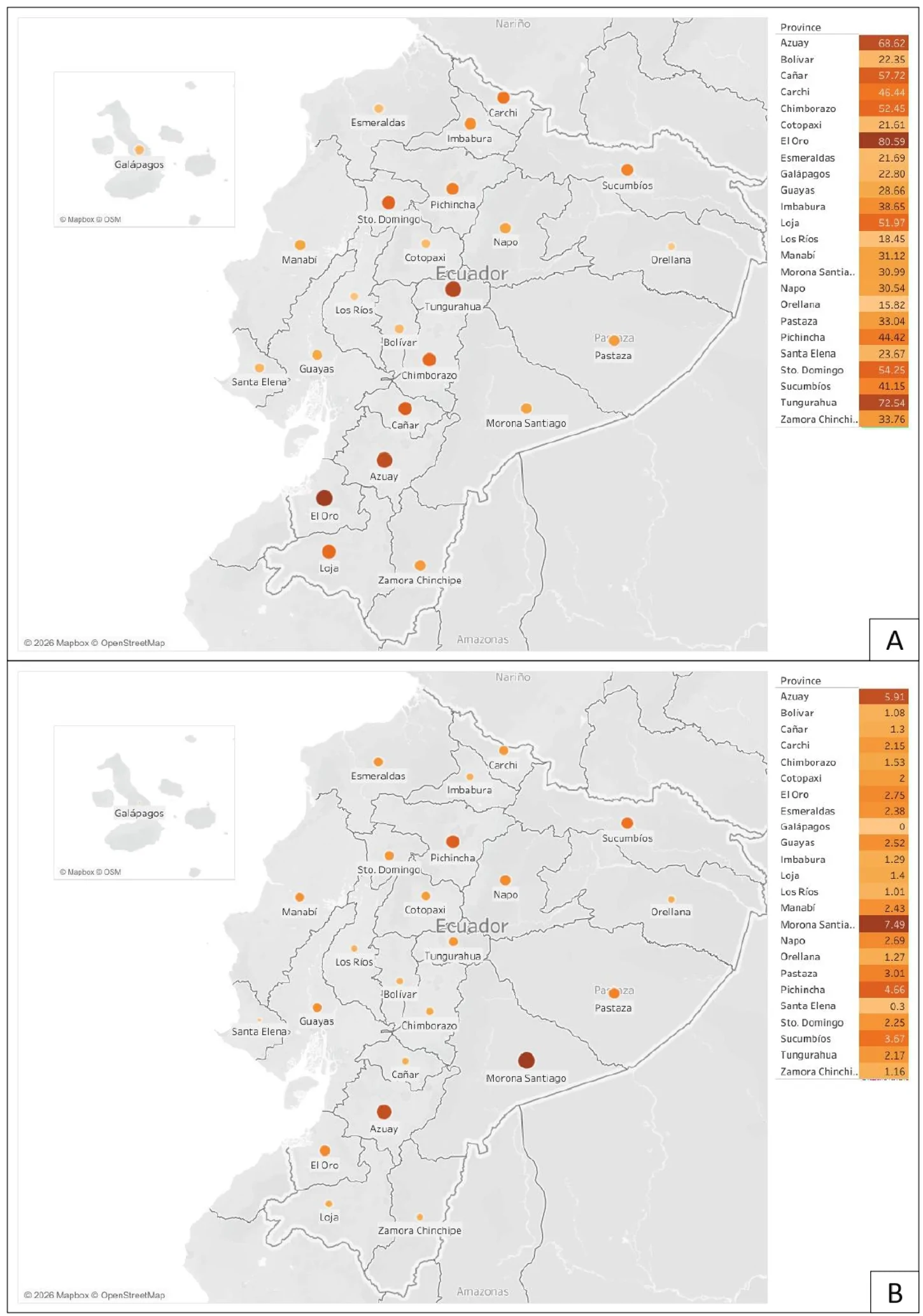
Mean crude acute respiratory infection (ARI) in-hospital mortality rates per 100,000 population by province, Ecuador, 2015–2023: (**A**) adults and (**B**) pediatric rates.

In pediatrics, the mean hospitalization rate was 520.01 per 100,000 individuals of pediatric ages, highest in Morona Santiago with 1158.74 hospitalizations per 100,000 individuals of pediatric ages, Pichincha with 779.03, and Tungurahua with 725.86 (Supplementary Table S3 and Figure 1). On the other hand, the pediatric mean in-hospital mortality rate was 2.77 per 100,000, with little variation from 2015 with 2.61 in-hospital deaths per 100,000 to 2.81 in 2020, 2.82 in 2021, 2.86 in 2022 and 2.77 in 2023 (Supplementary Table S4 and Figure 2). The highest mean pediatric in-hospital mortality rates were observed in the provinces of Morona Santiago (7.49), Azuay (5.91), Pichincha (4.66), Sucumbíos (3.67), and Pastaza (3.01).

Among adults, the mean hospitalization rate was 283.14 per 100,000; it was highest in Morona Santiago (536.90), Cañar (536.81), Azuay (493.46), Loja (424.13) and Tungurahua (421.42) (Supplementary Table S5 and Figure 1). The mean in-hospital mortality rate among adults was 39.44 per 100,000, with significant variations observed. The rate changed from 14.50 in-hospital deaths per 100,000 to 116.82 deaths in 2020 and 121.07 deaths in 2021 before subsequently decreasing in 2022, with 27.69 in-hospital deaths, and 2023, with 13.17 in-hospital deaths per 100,000. The mean adult in-hospital mortality rates between 2015 and 2023 were highest in El Oro (80.59), Tungurahua (72.54), Azuay (68.62), Cañar (57.72) and Santo Domingo de los Tsáchilas (54.25) (Supplementary Table S6 and Figure 2).

Annual hospitalization rates per 100,000 inhabitants varied markedly by acute respiratory infection subtype over the study period (Table 3). Acute upper respiratory infections remained relatively stable at around 32–36 per 100,000 in 2015–2019; they then dropped to 12.74 in 2020 and 9.42 in 2021 before rebounding to 31.13 in 2022 and 31.52 in 2023 (mean 28.00). Influenza surged from 0.65 in 2015 to a peak of 13.13 in 2018, declined to 0.32 in 2021, and then climbed again to 10.32 in 2023 (mean 5.54). Viral pneumonia rose steadily from 3.49 in 2015 to 7.89 in 2019, peaked at 20.43 in 2020, and stabilized around 15–22 thereafter (mean 10.74). Bacterial pneumonia peaked at 65.02 in 2018, fell to 16.19 in 2021, and then returned to 61.58 by 2023 (mean 46.51). “Other pneumonias due to other or unspecified organisms” climbed from 170.21 in 2015 to 194.74 in 2016, stayed above 140 through 2019, dipped to 59.23 in 2021, and rebounded to 183.60 in 2023 (mean 152.16). Acute bronchitis and bronchiolitis both followed similar U-shaped curves, with pre-pandemic rates of 22–24 and 11–14 dropping sharply in 2020–2021 (bronchitis: 6.68 in 2020, 5.79 in 2021; bronchiolitis: 4.99 in 2020, 4.24 in 2021) before rising again in 2023 (bronchitis 14.19; bronchiolitis 13.20; averages 16.93 and 11.40, respectively). COVID-19 emerged in 2020 with 322.19 hospitalizations per 100,000 inhabitants and 361.15 in 2021 before rapidly declining to 79.89 in 2022 and 12.99 hospitalizations in 2023 (mean 194.06).

### 3.3. Temporal Trends in Age-Adjusted ARI Rates

In the total population, the age-adjusted hospitalization rate increased from 281.67 per 100,000 population in 2015 to 520.01 in 2020, followed by a decline to 369.90 in 2022 and a subsequent increase to 395.15 in 2023. The age-adjusted in-hospital mortality rate showed a similar pattern, increasing from 11.30 per 100,000 population in 2015 to 83.91 in 2021, before declining to 9.82 in 2023. Age-adjusted ARI hospitalization and in-hospital mortality rates by calendar year and population groups are presented in Table 4.

Joinpoint regression analysis identified changes in temporal trends of age-adjusted rates. In the total population, age-adjusted hospitalization rates increased significantly from 2015 to 2021 (APC: 9.91%; 95% CI: 5.14 to 32.59), followed by a non-significant decline from 2021 to 2023 (APC: −12.35%; 95% CI: −27.53 to 4.38). The AAPC for the entire 2015–2023 period was 3.86% (95% CI: −0.21 to 9.96) (Table 4). Age-adjusted in-hospital mortality rates showed a significant estimated annual increase of 52.78% during 2015–2021 (APC: 52.78%; 95% CI: 37.38 to 153.45), followed by a significant annual decline of 71.28% (APC: −71.28%; 95% CI: −93.07 to −35.75) with an overall AAPC for the 2015–2023 period of 0.60% (95% CI: −25.39 to 39.31) (Table 4).

**Figure 3:**
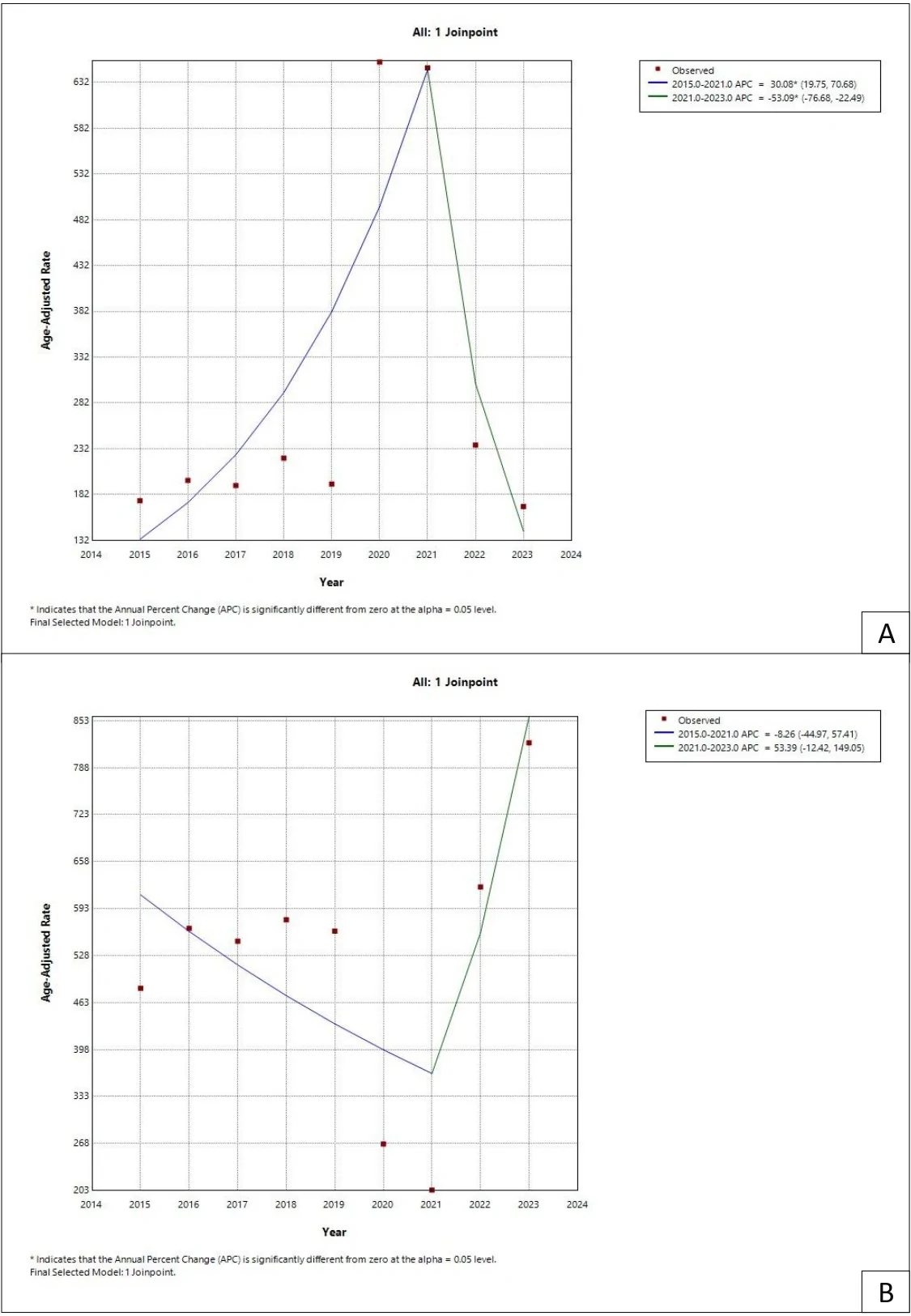
Age-adjusted acute respiratory infection hospitalization rates per 100,000 population and annual percentage changes: (**A**) adult hospitalization rates; (**B**) pediatric hospitalization rates.

**Figure 4:**
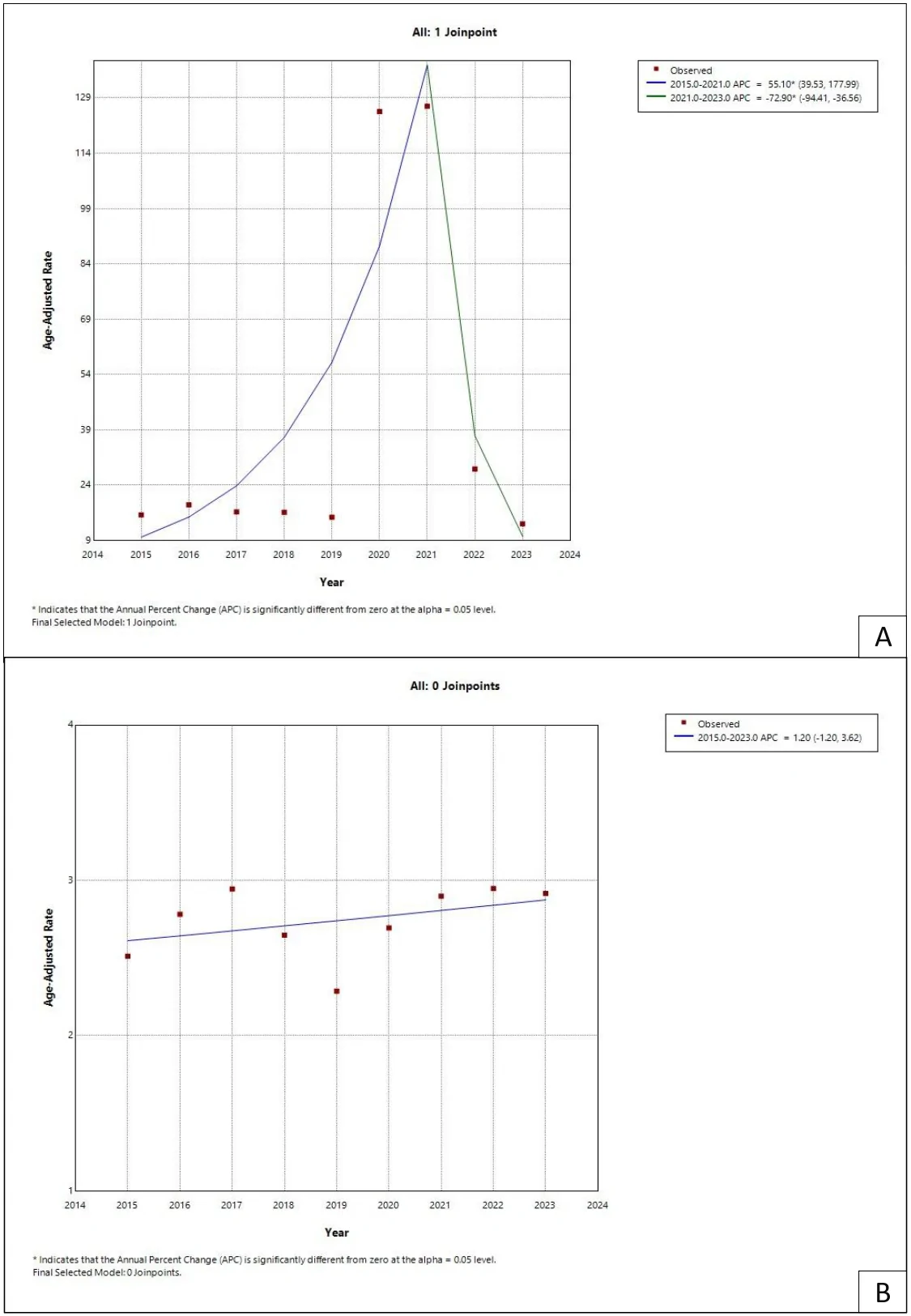
Age-adjusted acute respiratory infection in-hospital mortality rates per 100,000 population and annual percentage changes: (**A**) adults in-hospital mortality rates; (**B**) pediatric in-hospital mortality rates.

Temporal patterns differed between pediatric and adult populations (Table 4). Among adults, age-adjusted hospitalization rates showed a significant annual increase of 30.08% from 2015 to 2021 (APC: 30.08%; 95% CI: 19.75 to 70.68) and subsequently declined significantly from 2021 to 2023 with an annual decrease of 53.09% (APC: −53.09%; 95% CI: −76.68 to −22.49) (Figure 3). Adult in-hospital mortality showed a similar pattern, with a more pronounced significant annual increase of 55.10% during 2015–2021 (APC: 55.10%: 95% CI: 39.53 to 177.99) and significant decline from 2021 to 2023 (APC: −72.90%; 95% CI: −94.41 to −36.56) (Figure 4). In contrast, neither hospitalization nor in-hospital mortality rates among pediatric hospitalizations showed statistically significant temporal changes during the study period (Figure 3 and Figure 4).

### 3.4. Factors Associated with Acute Respiratory Infections In-Hospital Mortality

In the multivariable logistic regression model for the total sample (n = 576,195), age showed a strong age gradient in the odds of in-hospital mortality. Compared to individuals aged 20–29 years, the adjusted odds ratios (aOR) increased progressively, from 1.13 (95% CI: 1.01–1.26) in the 30–39 age group to 7.59 (95% CI: 6.92–8.32) in those aged ≥70 years. In contrast, children aged 0–4 years had substantially lower odds of in-hospital death (aOR: 0.29, 95% CI: 0.26–0.32), as did those aged 5–9 years (aOR: 0.22, 95% CI: 0.19–0.27) and 10–14 years (aOR: 0.49, 95% CI: 0.39–0.60) (Table 5).

When analyzing only the pediatric population, children aged 5–9 years had significantly lower odds of in-hospital mortality compared to those under 5 years (aOR: 0.77, 95% CI: 0.65–0.92), whereas those aged 10–19 years showed higher odds of mortality relative to the 0–4 age group (Table 6).

The adult-specific analysis mirrored the findings of the full sample, with all age groups over 29 years displaying increased odds of in-hospital mortality (Table 7).

Regarding sex, females were 22% less likely to die in-hospital than males in the total sample (aOR: 0.78, 95% CI: 0.76–0.79). However, in the pediatric subgroup, sex was not a statistically significant factor for in-hospital mortality, while in adults, females continued to show significantly lower odds (aOR: 0.77, 95% CI: 0.75–0.79) (Table 7).

With respect to ethnicity, when compared to Mestizos, Indigenous people (aOR: 0.73, 95% CI: 0.68–0.80), Montubios (aOR: 0.58, 95% CI: 0.43–0.77), Whites (aOR: 0.45, 95% CI: 0.40–0.50), and individuals from other ethnicities (aOR: 0.82, 95% CI: 0.78–0.87) had lower odds of in-hospital death in the total population. However, no significant associations by ethnicity were observed in the pediatric analysis. The adult subgroup mirrored the patterns seen in the full sample. The area of residence was not significantly associated with mortality in the full sample analysis. Nevertheless, pediatric cases living in rural areas had 20% higher odds of in-hospital death (aOR: 1.20, 95% CI: 1.06–1.37), while adults living in rural areas had slightly lower odds (aOR: 0.97, 95% CI: 0.94–0.99).

Private healthcare facilities were associated with significantly lower odds of in-hospital mortality across all analyses: total sample (aOR: 0.55, 95% CI: 0.53–0.56), pediatric group (aOR: 0.74, 95% CI: 0.65–0.84), and adult group (aOR: 0.54, 95% CI: 0.52–0.55).

In the fully adjusted model for all the study sample, nearly all types of acute respiratory infections were associated with increased odds of in-hospital mortality compared to acute upper respiratory infections, except for acute bronchitis which did not show significant associations. The highest odds were observed in hospitalizations with COVID-19 (aOR: 13.09, 95% CI: 10.61–16.15), viral pneumonia (aOR: 12.25, 95% CI: 9.84–15.26), bacterial pneumonia (aOR: 9.09, 95% CI: 7.37–11.22), pneumonia due to other or unspecified organisms (aOR: 7.49, 95% CI: 6.08–9.23), and influenza (aOR: 4.70, 95% CI: 3.58–6.16). These patterns were consistently observed in both pediatric and adult subgroup analyses, with bronchitis remaining non-significant across groups.

## 4. Discussion

The results of our study showed a marked difference between pediatric and adult populations in both hospitalization frequency and mortality associated with acute respiratory infections (ARI). At the national level, the mean hospitalization rate was 371.23 per 100,000 inhabitants, while the mean in-hospital mortality rate was 26.18 per 100,000. These findings demonstrate that ARI continue to represent a relevant burden for the Ecuadorian health system.

The Joinpoint regression further characterized the temporal trends, showing significant increases in age-adjusted ARI hospitalization and in-hospital mortality rates in the total population from 2015 to 2021, followed by a significant decline in in-hospital mortality from 2021 to 2023. The overall average annual percentage changes for 2015–2023 period were not significant, indicating that the segmented changes observed during the study period did not translate into significant overall trend across the entire study period. However, temporal patterns differed by age group, with adults showing significant increases in both age-adjusted hospitalization and in-hospital mortality rates from 2015 to 2021, followed by significant declines from 2021 to 2023, whereas pediatric trends were not statistically significant. These changes coincided with the COVID-19 pandemic period, in which the emergence of SARS-CoV-2 altered the epidemiology of ARI, both through the direct burden of COVID-19 and through changes in the circulation of other respiratory viruses [2]. In Ecuador, COVID-19 reshaped the epidemiology of acute respiratory infections during the pandemic peak. Between 2020 and 2021, COVID-19 accounted for 70.7% of all ARI hospitalizations and 87.2% of ARI in-hospital deaths. This impact was particularly pronounced among adults. Although the burden of COVID-19 declined substantially during 2022–2023, it still accounted for 12.6% of ARI hospitalizations and 36.9% of ARI deaths during this period, indicating a persistent contribution to severe respiratory outcomes even after the pandemic peak. Furthermore, overall ARI hospitalization rates during 2022–2023 remained above pre-pandemic levels, consistent with reports of a partial recovery and reconfiguration of respiratory infection epidemiology across Latin America [5,20]. This suggests that the post-pandemic impact persists, potentially driven by respiratory sequelae, coinfections, and population-level immune weakening following prolonged confinement measures [21].

The geographic distribution of ARI hospitalizations and deaths was heterogeneous. Andean and Amazonian provinces such as Morona Santiago, Azuay, and Tungurahua exhibited hospitalization and mortality rates above the national average. These findings may be consistent with the potential influence of environmental factors such as temperature and altitude [22], as well as structural inequalities in access to hospital care [10]. Studies conducted in Peru and Bolivia have shown that altitude modifies the severity and clinical course of ARI, particularly affecting young children and older adults [23,24]. The identification of provinces with persistently high rates highlights the need for targeted interventions, including strengthened epidemiological surveillance, the increased availability of critical care beds, and timely access to oxygen therapy, especially in rural regions with logistical limitations.

The adult-predominant mortality burden observed in this study aligns with regional and global evidence indicating that chronic comorbidities, immune senescence, and delayed healthcare-seeking behavior substantially worsen outcomes of acute respiratory infections in older age groups [25,26]. In 2023, global lower respiratory infections mortality in children under five declined by more than 30% compared with 2010, with many countries meeting international mortality targets, while mortality among adults aged 70 years and older showed only marginal reductions [26]. This divergence reflects improvements in pediatric prevention and case management, contrasted with persistent biological and clinical vulnerability among older adults. Pediatric populations benefit from earlier clinical suspicion, lower thresholds for hospitalization, and highly protocolized management pathways for respiratory infections, which likely contribute to improved survival despite high admission rates [5]. In addition, indirect protection conferred by routine childhood immunization programs, particularly against severe bacterial respiratory infections, and earlier presentation to healthcare services facilitated by caregivers may play roles in the low mortality observed among younger children [27,28]. However, the high frequency of pediatric admissions may suggest ongoing shortcomings in primary prevention and persistent exposure to adverse housing and environmental conditions, which continue to facilitate respiratory infection transmission in early childhood [12]. Additionally, male sex was associated with higher odds of in-hospital death, a pattern that likely reflects hormonal, immunological, and behavioral differences, with men exhibiting higher exposure to behavioral risk factors such as smoking and occupational hazards, as well as a greater burden of chronic cardiopulmonary comorbidities that impair immune responses and reduce pulmonary reserve [29,30]. In pediatric populations, our results showed no significant sex-related differences in mortality odds, suggesting that disparities in respiratory infection outcomes by sex become more pronounced primarily in adulthood, a finding supported by global analyses reporting no meaningful sex differences in mortality from upper and lower respiratory infections among children [31].

Sociodemographic factors, particularly ethnicity and rural residence, were associated with in-hospital mortality and need careful interpretation. Although Indigenous and Montubio populations showed lower odds of death during hospitalization than Mestizo individuals, these estimates should be interpreted cautiously because some ethnicity categories were based on very small numbers of deaths and had wide confidence intervals. This finding is unlikely to represent a true survival advantage. Instead, evidence from Ecuador and other Latin American settings indicates that geographic isolation, transportation difficulties, delayed referrals, and limited access to higher-level hospitals may contribute to deaths before hospital admissions [11,22,32,33]. As a result, hospital-based analyses primarily reflect outcomes among those who are able to access services, often individuals with less severe disease or better access, which may produce selection effects in hospital-based mortality estimates and an apparent reduction in in-hospital mortality that does not necessarily indicate lower population-level risk [33,34].

This perspective also helps explain why patterns differed between children and adults. In pediatric populations, although inequities in access persist at the community level, early hospitalization and standardized care likely reduce outcome differences among hospitalized children, allowing severely ill children from different ethnic and geographic backgrounds to reach hospital services and attenuating ethnic differences once admitted. However, even after multivariable adjustment, rural residence remained associated with higher mortality odds among children, likely reflecting delayed presentation, longer travel times, and limited availability of nearby specialized pediatric services [32,33]. In contrast, among adults’ hospitalizations, both ethnic background and rural residence were more likely to influence whether patients reach hospital care at all. Many severely ill adults in rural or marginalized settings may experience barriers to hospital admissions; consequently, hospitalized patients may represent a selected subgroup with different levels of disease severity. This may partly reflect why the association between rural residence and mortality significantly weakened after multivariable adjustment in adult analyses and why lower in-hospital mortality among rural adults should not be interpreted as a protective effect. Overall, these findings suggest that differences in ARI outcomes may partly reflect barriers to timely care before hospitalization, with effects that are more evident among children and less visible among adults once hospital admission occurs.

The type of healthcare institution was also associated with in-hospital mortality. Private hospitals showed significantly lower mortality than public institutions. This pattern has previously been described in an epidemiological surveillance study in Brazil [35]. Possible explanations include differences in resource availability, lower bed occupancy, and shorter waiting times in the private sector [35]. However, given that most of the Ecuadorian population depends on the public healthcare system, reducing gaps in intensive care capacity and human resources remains a priority for improving ARI-related hospital outcomes.

Analysis of ARI subtypes supports the presence of age-related clinical severity, as the strength and clinical interpretation of these associations varied by age. Across all age groups, lower respiratory tract infections, including bacterial pneumonia, viral pneumonia, COVID-19, and other pneumonias, were consistently associated with the highest odds of in-hospital mortality, suggesting that lower respiratory tract involvement is an important factor of severe ARI outcomes. Unlike upper respiratory tract infections, which are generally self-limiting, pneumonias, particularly among older adults, continue to account for a substantial proportion of ARI-related hospital deaths in Latin America [10]. The high frequency of pneumonia coded under J16–J18 (pneumonia due to other specified infectious organisms and pneumonia due to unspecified organisms) warrants consideration. In Ecuador, pneumonia and lower respiratory infections are commonly diagnosed on clinical characteristics and supported by chest radiography, whereas microbiological confirmation is less frequent outside intensive care units and tertiary healthcare centers [36]. Differences in diagnostic and coding practices across hospitals may also contribute to the use of nonspecific pneumonia codes [36]. Therefore, the high frequency of J16-J18 in the present study should not be interpreted as evidence of a predominant unidentified pathogen, but rather as potentially reflecting the diagnostic and coding characteristics of routine hospital-based surveillance.

In pediatric hospitalizations, overall mortality was low, but progression to lower respiratory tract disease markedly increased the odds of death, with pneumonias and bronchiolitis emerging as indicators of severe illness. Although bronchiolitis is common in early childhood and usually self-limited, hospitalization likely identifies a subset of infants with greater vulnerability [37], which may help explain the higher odds of in-hospital death compared with upper respiratory infections. Nonetheless, its overall contribution to pediatric mortality remained limited, consistent with children’s high physiological resilience.

In contrast, bronchiolitis in adults was associated with substantially higher mortality odds than in children. In adult populations, bronchiolitis is uncommon and typically reflects severe disease occurring in the context of chronic lung conditions, immunosuppression, or severe viral infection, representing a marker of critical lower airway involvement rather than a benign condition [38]. Consequently, hospitalized adult cases may represent a selected subgroup with greater clinical vulnerability and disease severity, and may be associated with greater mortality risk. Similarly, the increased lethality associated with COVID-19 and pneumonias among adults persisted after multivariable adjustment, suggesting the influence of vulnerability mechanisms not fully captured in this study, such as a higher burden of chronic comorbidities, reduced physiological reserve, immune senescence, and greater disease severity at presentation. Together, these findings indicate that while the spectrum of severe ARIs is broadly similar across age groups, the prognostic significance of specific infection subtypes is strongly shaped by life-course–related vulnerability.

From a public health perspective, these results highlight the urgency of implementing differentiated strategies. In pediatric populations, priority should be placed on primary prevention, including immunization, improvements in environmental sanitation, a reduction in household crowding, and promotion of breastfeeding [28]. Among adults and older adults, interventions should focus on comorbidity management, annual vaccination against influenza and pneumococcus, and early access to respiratory support therapies [39]. The experience of the pandemic peak demonstrated the fragility of hospital systems in the face of abrupt increases in demand, underscoring the importance of strengthening primary care and hospital response capacity [40,41].

Several limitations of this study should be acknowledged. First, the analysis relied on secondary administrative data, which are subject to potential misclassification, underreporting, and variability in diagnostic coding practices across institutions and over time, particularly during periods of health system strain such as the COVID-19 pandemic. In particular, the introduction of ICD-10 codes U07.1-U07.2 may have affected the classification of respiratory diagnoses during the study period. Although our review of the INEC databases analyzed in this study found no alternative COVID-19 specific codes and U07.1-U07.2 remained the codes used to identify COVID-19 cases in the post-2020 databases, the introduction of these codes may have altered the distribution of respiratory diagnoses compared with the pre-pandemic period. Therefore, temporal changes in ARI subtypes, particularly across the 2020–2021 transition, should be interpreted with caution. In addition, key clinical variables including disease severity at admission, intensive care unit utilization, laboratory findings, vaccination status, and detailed comorbidity profiles were not available, limiting the ability of the multivariable regression models to fully account for clinical complexity and residual confounding.

Although temporal trends in annual ARI hospitalization and in-hospital mortality rates were assessed using Joinpoint regression, within-year seasonal variation was not specifically evaluated because it was beyond the predefined scope of this study and represents an important area for future research.

The publicly available INEC database does not contain a unique patient identifier, which made it difficult to identify repeated hospitalizations or readmissions by the same individual or distinguish them from hospitalizations involving different individuals. Consequently, the analysis was conducted at the hospitalization discharge record level, and the possibility of correlated observations resulting from repeated admissions cannot be excluded.

Information on pre-hospital care, referral delays, and deaths occurring outside hospital settings was also not captured. This limitation is particularly relevant when interpreting associations related to sociodemographic factors such as ethnicity and rural residence, as it may contribute to selection effects in hospital-based mortality estimates. Because pre-hospital mortality and referral delays were not measured in this study, these factors remain plausible explanations rather than directly demonstrated pathways.

Furthermore, the COVID-19 pandemic represents an important external influence that likely affected healthcare utilization and clinical outcomes through disruptions in access and diagnostic processes. To partially account for these time-dependent effects, year of hospital admission was included as a covariate in the multivariable analyses. Finally, the cross-sectional design of the study precludes assessment of risk, and all associations should be interpreted as correlational. Despite these limitations, this study has several notable strengths, including its large, nationwide administrative hospital discharge dataset, extended observation period, and consistent findings across multiple analytical strata.

## 5. Conclusions

This nationwide analysis shows that acute respiratory infections (ARIs) represent a substantial and unequal burden on the Ecuadorian health system, with a clear divergence between hospitalization patterns and mortality across age groups. While children account for most of these admissions, in-hospital deaths are overwhelmingly concentrated among adults, particularly older adults. Temporal trends also differed by age group, with significant increases in age-adjusted ARI hospitalization and in-hospital mortality rates among adults from 2015 to 2021, followed by significant declines from 2021 to 2023, whereas pediatric trends were not significant. These findings suggest that hospitalization volume alone does not fully capture the burden of ARI-related in-hospital mortality. Mortality patterns varied according to age, ARI subtype and sociodemographic characteristics, while observed geographic and sociodemographic differences may partly reflect selection effects related to access to hospital care. Reducing ARI mortality in Ecuador will require age-differentiated strategies that extend beyond hospital-based care. Strengthening prevention and early detection in childhood, improving access and referral pathways in rural areas, and prioritizing adult and older adult risk reduction through chronic disease management and vaccination may help mitigate future respiratory disease burden. Addressing the persistent impact of ARIs will require integrated, equity-oriented approaches aligned with population risk profiles to strengthen health system resilience in the post-pandemic era.

## Figures and Tables

**Table 1:** General characteristics of acute respiratory infections hospitalizations in Ecuador between 2015 and 2023.

Variables	Pediatric Population (n = 295,225)				Adult Population (n = 280,970)				Total (n = 576,195)	
	Alive (n = 293,650)		Dead (n = 1575)		Alive (n = 241,497)		Dead (n = 39,473)			
	n	%	n	%	n	%	n	%	n	%
**Age in years: median (IQR)**	1 (0–4)		1 (0–5)		63 (47–78)		72 (61–83)		14 (1–64)	
**Age groups**	***p*** ** ≤ 0.001**				***p*** ** ≤ 0.001**					
0–4 years	233,697	99.51%	1153	0.49%	N/A		N/A		234,850	40.76%
5–9 years	41,387	99.65%	147	0.35%	N/A		N/A		41,534	7.21%
10–14 years	12,233	99.12%	109	0.88%	N/A		N/A		12,342	2.14%
15–19 years	6333	97.45%	166	2.55%	N/A		N/A		6499	1.13%
20–29 years	N/A		N/A		15,773	96.90%	504	3.10%	16,277	2.82%
30–39 years	N/A		N/A		23,823	95.59%	1098	4.41%	24,921	4.33%
40–49 years	N/A		N/A		28,372	92.45%	2316	7.55%	30,688	5.33%
50–59 years	N/A		N/A		37,291	88.29%	4948	11.71%	42,239	7.33%
60–69 years	N/A		N/A		41,073	83.35%	8203	16.65%	49,276	8.55%
≥70 years	N/A		N/A		95,165	80.94%	22,404	19.06%	117,569	20.40%
**Sex**	*p* = 0.785				***p*** ** ≤ 0.001**					
Male	160,641	99.46%	867	0.54%	124,459	84.05%	23,626	15.95%	309,593	53.73%
Female	133,009	99.47%	708	0.53%	117,038	88.07%	15,847	11.93%	266,602	46.27%
**Ethnicity**	***p*** ** ≤ 0.001**				***p*** ** ≤ 0.001**					
Mestizo	235,599	99.44%	1319	0.56%	189,035	85.56%	31,896	14.44%	457,849	79.46%
Indigenous	15,529	99.30%	109	0.70%	6142	91.28%	587	8.72%	22,367	3.88%
Black/Afro	1774	99.61%	7	0.39%	1013	86.43%	159	13.57%	2953	0.51%
Montubio	889	99.89%	1	0.11%	591	91.49%	55	8.51%	1536	0.27%
White	1458	99.93%	1	0.07%	3859	91.68%	350	8.32%	5668	0.98%
Other	7937	99.37%	50	0.63%	12,164	86.53%	1893	13.47%	22,044	3.83%
Unknown	30,464	99.71%	88	0.29%	28,693	86.36%	4533	13.64%	63,778	11.07%
**Area of residence**	***p*** ** ≤ 0.001**				***p*** ** ≤ 0.001**					
Urban	247,788	99.50%	1254	0.50%	204,304	85.84%	33,688	14.16%	487,034	84.53%
Rural	45,862	99.30%	321	0.70%	37,193	86.54%	5785	13.46%	89,161	15.47%
**Healthcare sector**	***p*** ** ≤ 0.001**				***p*** ** ≤ 0.001**					
Public	210,956	99.41%	1245	0.59%	169,075	83.59%	33,203	16.41%	414,479	71.93%
Private	82,694	99.60%	330	0.40%	72,422	92.03%	6270	7.97%	161,716	28.07%
**Types of respiratory infections**	***p*** ** ≤ 0.001**				***p*** ** ≤ 0.001**					
Acute upper respiratory infections	33,693	99.90%	33	0.10%	9316	99.38%	58	0.62%	43,100	7.48%
Influenza	5115	99.57%	22	0.43%	3384	96.80%	112	3.20%	8633	1.50%
Viral pneumonia	11,258	99.64%	41	0.36%	4639	83.27%	932	16.73%	16,870	2.93%
Bacterial pneumonia	39,472	99.20%	319	0.80%	28,194	87.99%	3850	12.01%	71,835	12.47%
Other pneumonias due to other or unspecified organisms	143,852	99.37%	919	0.63%	80,853	90.22%	8767	9.78%	234,391	40.68%
Acute bronchitis	19,808	99.90%	20	0.10%	6067	99.33%	41	0.67%	25,936	4.50%
Acute bronchiolitis	17,189	99.84%	27	0.16%	350	97.77%	8	2.23%	17,574	3.05%
Unspecified acute lower respiratory infection	15,793	99.67%	53	0.33%	3086	94.95%	164	5.05%	19,096	3.31%
Suppurative and necrotic conditions of lower respiratory tract	475	97.74%	11	2.26%	1596	92.74%	125	7.26%	2207	0.38%
COVID-19	6995	98.16%	130	1.84%	104,012	80.36%	25,416	19.64%	136,553	23.70%
**Year of admission**	***p*** ** ≤ 0.001**				***p*** ** ≤ 0.001**					
2015	31,820	99.48%	166	0.52%	14,602	91.04%	1437	8.96%	48,025	8.33%
2016	37,312	99.51%	184	0.49%	16,860	90.70%	1728	9.30%	56,084	9.73%
2017	36,089	99.46%	195	0.54%	16,937	91.38%	1597	8.62%	54,818	9.51%
2018	38,004	99.54%	175	0.46%	20,437	92.60%	1633	7.40%	60,249	10.46%
2019	36,923	99.59%	151	0.41%	18,273	92.18%	1551	7.82%	56,898	9.87%
2020	17,420	98.99%	178	1.01%	55,705	80.98%	13,083	19.02%	86,386	14.99%
2021	12,154	98.56%	177	1.44%	57,383	80.70%	13,725	19.30%	83,439	14.48%
2022	37,099	99.52%	178	0.48%	23,567	88.10%	3182	11.90%	64,026	11.11%
2023	46,829	99.64%	171	0.36%	17,733	92.02%	1537	7.98%	66,270	11.50%

Note: IQR, interquartile range; n, frequency; %, percentage; N/A, not applicable. *p*-values were calculated with the χ^2^ test or Fisher’s exact for comparing categorical variables. Statistically significant effects (*p* < 0.05) are given in bold.

**Table 2:** Acute respiratory infections hospitalization rates and in-hospital mortality rates by provinces and by calendar years, per 100,000 inhabitants for the total study sample.

Acute Respiratory Infections Hospitalization Rates by Provinces and Years per 100,000 Inhabitants										
Provinces	2015	2016	2017	2018	2019	2020	2021	2022	2023	Mean Rates 2015–2023
Azuay	454.79	499.86	480.78	539.82	474.98	726.87	671.73	574.89	611.83	559.51
Bolívar	180.67	238	241.04	262.13	253.53	415.05	440.93	303.58	389.12	302.67
Cañar	457.91	591.36	555.27	716.9	606.81	614.65	582.03	649.88	621.82	599.62
Carchi	305.76	327.3	380.91	368.91	354	429.73	543.18	343.52	306.46	373.31
Cotopaxi	296.04	296.99	283.88	304.73	263.21	407.1	384.56	314.41	313.12	318.23
Chimborazo	399.57	522.88	483.13	460.49	425.25	570.86	531.89	460.98	470.28	480.59
El Oro	411.3	504.26	428.36	379.86	325.97	522.2	567.31	386.53	317.4	427.02
Esmeraldas	148.07	156.32	182.17	258.83	221.87	228.29	194.5	166.7	170.11	191.87
Guayas	258.75	270.16	229	226.25	264.67	331.44	333.71	242.58	231.95	265.39
Imbabura	315.19	342.63	396.52	414.43	350.73	466.75	555.97	420.86	422.28	409.48
Loja	331.13	351.43	392.51	455.75	407.67	514.61	645.28	525.2	535.75	462.15
Los Ríos	208.35	236.7	252.07	249.68	199.61	274.38	272.77	228.84	206.81	236.58
Manabí	230.64	246.38	258.6	272.14	242.36	287.52	283.89	233.08	238.38	254.78
Morona Santiago	791.6	924.42	1005.04	1117.31	871.69	762.05	679.56	685.37	754.25	843.48
Napo	311.57	339.9	290.07	259.39	299.62	379.56	329.36	254.51	300.1	307.12
Pastaza	438.14	513.62	414.89	411.33	437.24	391.08	498.36	370.53	389.54	429.41
Pichincha	292.54	392.92	392.57	457.55	409.8	843.69	780.59	542.46	608.11	524.47
Tungurahua	363.25	428.8	403.56	499.11	473.94	840.89	666.79	511.17	574.94	529.16
Zamora Chinchipe	287.06	320.99	385.47	517.86	441.15	422.15	386.26	489.14	574.93	425
Galápagos	197.92	279.2	308.43	308.46	280.41	312.04	387.68	240.98	238.19	283.7
Sucumbíos	447.72	367.56	310.81	339.8	304.71	449.17	384.16	235.5	237.93	341.93
Orellana	179.47	237.19	218.95	151.59	138.59	242.37	250.72	174.04	181.47	197.15
Santo Domingo de los Tsáchilas	294.37	314.12	268.97	330.86	269.31	532.24	477.56	379.8	313.74	353.44
Santa Elena	215.27	257.06	282.07	276.91	235.69	303.69	190.82	215.19	220.29	244.11
Total	295.19	339.24	326.44	352.79	327.81	492.89	473.7	361.42	371.58	371.23
**Acute respiratory infections in-hospital mortality rates by provinces and years per 100,000 inhabitants**										
**Provinces**	**2015**	**2016**	**2017**	**2018**	**2019**	**2020**	**2021**	**2022**	**2023**	**Mean rates 2015–2023**
Azuay	18.07	22.91	25.75	21.77	19.87	115.92	130.42	41.27	23.18	46.57
Bolívar	6.18	8.55	6.66	3.81	6.22	42.32	43.08	6.32	5.37	14.28
Cañar	11.47	14.38	17.71	22.3	15.97	111.34	86.63	22.31	15.58	35.3
Carchi	16.54	12.93	8.87	14.25	14.7	85.51	107.98	11.06	9.49	31.26
Cotopaxi	9.69	6.44	5.92	9.1	6.85	34.99	35.51	12.08	8.25	14.31
Chimborazo	8.89	14.46	14.01	11.79	13.4	114.17	101.66	21.7	8.76	34.32
El Oro	19.75	18.97	19.79	11.97	10.98	160.73	186.62	41.27	12.35	53.6
Esmeraldas	4.93	5.4	6.04	4.97	5.1	48.4	37.08	5.71	5.51	13.68
Guayas	10.92	13.88	11.68	9.23	10.38	51.94	42.4	12.68	8.29	19.05
Imbabura	5.09	7.87	8.85	8.96	5.69	75.01	92.49	15.43	6.94	25.15
Loja	12.94	12.09	9.65	11.05	10.44	74.38	128.69	29.42	14.7	33.71
Los Ríos	5.88	4.98	5.11	5.77	2.99	31.83	31.62	11.23	7.71	11.9
Manabí	4.65	9.28	5.9	5.92	6.31	59.44	68.46	17.66	8.37	20.66
Morona Santiago	14.36	12.36	17.6	16.7	10.56	41.99	36.71	16.55	10.35	19.69
Napo	6.68	4.91	2.41	3.15	8.52	69.36	53.39	11.13	4.39	18.22
Pastaza	0	8.91	7.72	10.38	12.02	52.51	72.71	11.31	3.42	19.89
Pichincha	9.01	10.68	10.45	13.07	10.76	94.57	106.14	23.61	9.51	31.98
Tungurahua	20.3	14.85	14.89	20.46	16.76	162.49	153.23	21.26	9.37	48.18
Zamora Chinchipe	7.68	8.5	8.36	11.87	4.49	49.67	58.82	17.38	13.75	20.06
Galápagos	11	7.25	0	0	3.51	48.54	54.89	3.39	10.06	15.4
Sucumbíos	6.1	4.51	7.41	15.13	7.25	73.66	85.69	19.3	10.18	25.47
Orellana	3.77	1.22	2.39	1.17	2.85	33.51	30.66	5.37	3.17	9.34
Santo Domingo de los Tsáchilas	5.54	7.11	4.53	8.11	11.01	104.62	117.08	31.15	15.62	33.86
Santa Elena	5.07	7.42	4.56	6.31	3.36	53.76	40.58	9.77	2.74	14.84
Total	9.85	11.57	10.67	10.59	9.81	75.66	78.92	18.97	9.58	26.18

**Table 3:** Acute respiratory infections hospitalization rates and in-hospital mortality rates by type of acute respiratory infection and by calendar years, per 100,000 inhabitants for the total study sample.

Hospitalization Rates by Types of Acute Respiratory Infections per 100,000 Inhabitants										
Type of Respiratory Infection	2015	2016	2017	2018	2019	2020	2021	2022	2023	Mean Rates 2015–2023
Acute upper respiratory infections	33.61	35.78	34.01	32.19	31.56	12.74	9.42	31.13	31.52	28
Influenza	0.65	2.95	4.25	13.13	9.53	2.96	0.32	5.72	10.32	5.54
Viral pneumonia	3.49	4.65	5.23	7.04	7.89	20.43	10.6	15.2	22.1	10.74
Bacterial pneumonia	41.98	50.34	52.45	65.02	61.87	27.46	16.19	41.7	61.58	46.51
Other pneumonias due to other or unspecified organisms	170.21	194.74	179.84	184.59	168.6	86.76	59.23	141.87	183.6	152.16
Acute bronchitis	24.4	24.89	22.7	21.49	16.82	6.68	5.79	15.41	14.19	16.93
Acute bronchiolitis	11.8	14.48	14.51	13.3	14.33	4.99	4.24	11.79	13.2	11.4
Unspecified acute lower respiratory infection	7.66	10.15	12.16	14.62	15.77	7.54	5.57	16.91	20.16	12.28
Suppurative and necrotic conditions of lower respiratory tract	1.38	1.26	1.29	1.41	1.43	1.15	1.19	1.79	1.91	1.42
COVID-19	0	0	0	0	0	322.19	361.15	79.89	12.99	194.06
Total	295.19	339.24	326.44	352.79	327.81	492.89	473.7	361.42	371.58	371.23
**In** **-hospital mortality rates by types of acute respiratory infections per 100,000 inhabitants**										
**Type of respiratory infection**	**2015**	**2016**	**2017**	**2018**	**2019**	**2020**	**2021**	**2022**	**2023**	**Mean rates 2015–2023**
Acute upper respiratory infections	0.02	0.07	0.07	0.02	0.05	0.07	0.09	0.07	0.07	0.06
Influenza	0.02	0.16	0.05	0.36	0.06	0.03	0.02	0.04	0.05	0.09
Viral pneumonia	0.06	0.07	0.03	0.11	0.16	3.59	1.13	0.27	0.13	0.62
Bacterial pneumonia	2.33	2.82	3.1	3.27	3.44	2.17	2.11	2.52	2.52	2.7
Other pneumonias due to other or unspecified organisms	7.2	8.13	7.12	6.57	5.87	5.79	4.16	5.86	5.89	6.29
Acute bronchitis	0.06	0.06	0.06	0.04	0.01	0.05	0.02	0.03	0.04	0.04
Acute bronchiolitis	0.02	0.05	0.02	0.01	0.02	0.01	0.07	0.01	0	0.02
Unspecified acute lower respiratory infection	0.07	0.14	0.13	0.12	0.11	0.29	0.1	0.14	0.15	0.14
Suppurative and necrotic conditions of lower respiratory tract	0.07	0.07	0.1	0.09	0.1	0.07	0.09	0.06	0.14	0.09
COVID-19	0	0	0	0	0	63.59	71.15	9.96	0.58	36.32
Total	9.85	11.57	10.67	10.59	9.81	75.66	78.92	18.97	9.58	26.18

**Table 4:** Age-adjusted acute respiratory infection hospitalization and in-hospital mortality rates per 100,000 population, with annual percentage changes (APCs) and average annual percentage changes (AAPCs), Ecuador, 2015–2023.

Years	Age Adjusted Hospitalization Rates			Age Adjusted In-Hospital Mortality Rates		
	Total Population	Pediatric Population	Adult Population	Total Population	Pediatric Population	Adult Population
2015	281.67	482.55	175.35	11.30	2.51	15.95
2016	324.89	565.68	197.45	13.15	2.78	18.64
2017	315.07	547.87	191.86	11.98	2.94	16.77
2018	344.93	577.40	221.90	11.78	2.65	16.62
2019	320.97	561.82	193.50	10.78	2.29	15.28
2020	520.01	267.04	653.90	82.87	2.69	125.30
2021	493.78	203.43	647.45	83.91	2.90	126.78
2022	369.90	622.77	236.07	19.57	2.95	28.36
2023	395.15	822.50	168.99	9.82	2.92	13.47
Annual percentage change (APC) and average annual percentage changes (AAPC) and their 95% confidence intervals (95% CI)						
	**APC**	**APC lower CI**	**APC Upper CI**	**AAPC**	**AAPC lower CI**	**AAPC Upper CI**
**Acute respiratory infections hospitalizations in total population**						
**2015–2021**	9.91	5.14	32.59	3.86	−0.21	9.96
**2021–2023**	−12.35	−27.53	4.38			
**Acute respiratory infections hospitalizations in pediatric population**						
**2015–2021**	−8.26	−44.97	57.41	4.32	−9.07	16.71
**2021–2023**	53.39	−12.42	149.05			
**Acute respiratory infections hospitalizations in adult population**						
**2015–2021**	30.08 *	19.75	70.68	0.81	−13.44	18.87
**2021–2023**	−53.09 *	−76.68	−22.49			
**Acute respiratory infections in-hospital deaths in total population**						
**2015–2021**	52.78 *	37.38	153.45	0.60	−25.39	39.31
**2021–2023**	−71.28 *	−93.07	−35.75			
**Acute respiratory infections in-hospital deaths in pediatric population**						
**2015–2023**	1.20	−1.20	3.62	1.20	−1.20	3.62
**Acute respiratory infections in-hospital deaths in adult population**						
**2015–2021**	55.10 *	39.53	177.99	0.27	−28.00	44.33
**2021–2023**	−72.90 *	−94.41	−36.56			

* The annual percentage change (APC) or the average APC (AAPC) is significantly different from zero at the alpha = 0.05 level.

**Table 5:** Crude odds ratios and adjusted odds ratios for factors associated with acute respiratory infections in-hospital mortality for the total study sample.

Variables	Total Population			
	OR (95% CI)	*p*-Value	aOR (95% CI)	*p*-Value
**Age groups**				
0–4 years	**0.15 (0.14–0.17)**	***p*** ** ≤ 0.001**	**0.29 (0.26–0.32)**	***p*** ** ≤ 0.001**
5–9 years	**0.11 (0.09–0.13)**	***p*** ** ≤ 0.001**	**0.22 (0.19–0.27)**	***p*** ** ≤ 0.001**
10–14 years	**0.28 (0.23–0.34)**	***p*** ** ≤ 0.001**	**0.49 (0.39–0.60)**	***p*** ** ≤ 0.001**
15–19 years	**0.82 (0.69–0.98)**	**0.0290**	1.09 (0.91–1.31)	0.335
20–29 years	Reference		Reference	
30–39 years	**1.44 (1.30–1.61)**	***p*** ** ≤ 0.001**	**1.13 (1.01–1.26)**	**0.033**
40–49 years	**2.55 (2.32–2.82)**	***p*** ** ≤ 0.001**	**1.76 (1.59–1.95)**	***p*** ** ≤ 0.001**
50–59 years	**4.15 (3.78–4.56)**	***p*** ** ≤ 0.001**	**2.82 (2.57–3.11)**	***p*** ** ≤ 0.001**
60–69 years	**6.25 (5.70–6.85)**	***p*** ** ≤ 0.001**	**4.56 (4.15–5.01)**	***p*** ** ≤ 0.001**
≥70 years	**7.37 (6.74–8.06)**	***p*** ** ≤ 0.001**	**7.59 (6.92–8.32)**	***p*** ** ≤ 0.001**
**Sex**				
Male	Reference		Reference	
Female	0.77 (0.76–0.79)	***p*** ** ≤ 0.001**	0.78 (0.76–0.79)	***p*** ** ≤ 0.001**
**Ethnicity**				
Mestizo	Reference		Reference	
Indigenous	**0.41 (0.38–0.44)**	***p*** ** ≤ 0.001**	**0.73 (0.68–0.80)**	***p*** ** ≤ 0.001**
Black/Afro	**0.76 (0.65–0.89)**	***p*** ** ≤ 0.001**	1.06 (0.89–1.26)	0.517
Montubio	**0.48 (0.37–0.63)**	***p*** ** ≤ 0.001**	**0.58 (0.43–0.77)**	***p*** ** ≤ 0.001**
White	**0.84 (0.76–0.94)**	**0.002**	**0.45 (0.40–0.50)**	***p*** ** ≤ 0.001**
Other	**1.24 (1.18–1.30)**	***p*** ** ≤ 0.001**	**0.82 (0.78–0.87)**	***p*** ** ≤ 0.001**
Unknown	1.00 (0.97–1.03)	0.913	**0.78 (0.75–0.81)**	***p*** ** ≤ 0.001**
**Area of residence**				
Urban	Reference		Reference	
Rural	**0.95 (0.92–0.98)**	***p*** ** ≤ 0.001**	0.98 (0.95–1.01)	0.161
**Healthcare sector**				
Public	Reference		Reference	
Private	**0.47 (0.46–0.48)**	***p*** ** ≤ 0.001**	**0.55 (0.53–0.56)**	***p*** ** ≤ 0.001**
**Type of respiratory infection**				
Acute upper respiratory infections	Reference		Reference	
Influenza	**7.45 (5.70–9.73)**	***p*** ** ≤ 0.001**	**4.70 (3.58–6.16)**	***p*** ** ≤ 0.001**
Viral pneumonia	**28.93 (23.32–35.89)**	***p*** ** ≤ 0.001**	**12.25 (9.84–15.26)**	***p*** ** ≤ 0.001**
Bacterial pneumonia	**29.12 (23.65–35.85)**	***p*** ** ≤ 0.001**	**9.09 (7.37–11.22)**	***p*** ** ≤ 0.001**
Other pneumonias due to other or unspecified organisms	**20.37 (16.57–25.05)**	***p*** ** ≤ 0.001**	**7.49 (6.08–9.23)**	***p*** ** ≤ 0.001**
Acute bronchitis	1.11 (0.81–1.54)	0.514	0.78 (0.56–1.08)	0.14
Acute bronchiolitis	0.94 (0.64–1.39)	0.769	**1.94 (1.31–2.88)**	***p*** ** ≤ 0.001**
Unspecified acute lower respiratory infection	**5.43 (4.25–6.94)**	***p*** ** ≤ 0.001**	**4.22 (3.29–5.41)**	***p*** ** ≤ 0.001**
Suppurative and necrotic conditions of lower respiratory tract	**31.04 (23.71–40.62)**	***p*** ** ≤ 0.001**	**7.6 (5.77–10.00)**	***p*** ** ≤ 0.001**
COVID-19	**108.82 (88.55–133.74)**	***p*** ** ≤ 0.001**	**13.09 (10.61–16.15)**	***p*** ** ≤ 0.001**
**Year of admission**				
2015	Reference		Reference	
2016	1.02 (0.96–1.09)	0.515	**1.07 (1–1.15)**	**0.05**
2017	0.98 (0.91–1.05)	0.548	0.96 (0.89–1.03)	0.259
2018	**0.90 (0.84–0.96)**	**0.002**	**0.85 (0.79–0.91)**	***p*** ** ≤ 0.001**
2019	**0.90 (0.84–0.96)**	**0.002**	**0.84 (0.78–0.91)**	***p*** ** ≤ 0.001**
2020	**5.25 (4.98–5.54)**	***p*** ** ≤ 0.001**	**1.72 (1.61–1.83)**	***p*** ** ≤ 0.001**
2021	**5.79 (5.49–6.10)**	***p*** ** ≤ 0.001**	**1.77 (1.66–1.89)**	***p*** ** ≤ 0.001**
2022	**1.60 (1.51–1.70)**	***p*** ** ≤ 0.001**	1.01 (0.95–1.08)	0.701
2023	**0.77 (0.71–0.82)**	***p*** ** ≤ 0.001**	**0.81 (0.75–0.87)**	***p*** ** ≤ 0.001**

Note: OR, odds ratio; aOR, adjusted odds ratio; 95% CIs, 95% confidence intervals. Statistically significant effects (*p* < 0.05) are presented in bold. Odds ratios adjusted for age groups, sex, ethnicity, area of residence, healthcare sector, type of acute respiratory infection, and year of hospital admission.

**Table 6:** Crude odds ratios and adjusted odds ratios for factors associated with acute respiratory infections in-hospital mortality for the pediatric sample.

Variables	Pediatric Population			
	OR (95% CI)	*p*-Value	aOR (95% CI)	*p*-Value
**Age groups**				
0–4 years	Reference		Reference	
5–9 years	**0.72 (0.61–0.85)**	***p*** ** ≤ 0.001**	**0.77 (0.65–0.92)**	**0.004**
10–14 years	**1.80 (1.48–2.20)**	***p*** ** ≤ 0.001**	**1.52 (1.24–1.86)**	***p*** ** ≤ 0.001**
15–19 years	**5.31 (4.50–6.26)**	***p*** ** ≤ 0.001**	**3.57 (2.98–4.27)**	***p*** ** ≤ 0.001**
20–29 years	N/A		N/A	
30–39 years	N/A		N/A	
40–49 years	N/A		N/A	
50–59 years	N/A		N/A	
60–69 years	N/A		N/A	
≥70 years	N/A		N/A	
**Sex**				
Male	Reference		Reference	
Female	0.99 (0.89–1.09)	0.807	0.96 (0.87–1.06)	0.409
**Ethnicity**				
Mestizo	Reference		Reference	
Indigenous	**1.25 (1.03–1.52)**	**0.024**	1.04 (0.85–1.28)	0.671
Black/Afro	0.70 (0.33–1.48)	0.356	0.63 (0.30–1.34)	0.234
Montubio	0.20 (0.03–1.43)	0.109	0.20 (0.03–1.40)	0.104
White	**0.12 (0.02–0.87)**	**0.036**	0.16 (0.02–1.13)	0.067
Other	1.12 (0.85–1.49)	0.417	1.14 (0.85–1.51)	0.38
Unknown	**0.52 (0.42–0.64)**	***p*** ** ≤ 0.001**	**0.57 (0.46–0.72)**	***p*** ** ≤ 0.001**
**Area of residence**				
Urban	Reference		Reference	
Rural	**1.38 (1.22–1.56)**	***p*** ** ≤ 0.001**	**1.20 (1.06–1.37)**	**0.005**
**Healthcare sector**				
Public	Reference		Reference	
Private	**0.68 (0.60–0.76)**	***p*** ** ≤ 0.001**	**0.74 (0.65–0.84)**	***p*** ** ≤ 0.001**
**Type of respiratory infections**				
Acute upper respiratory infections	Reference		Reference	
Influenza	**4.39 (2.56–7.54)**	***p*** ** ≤ 0.001**	**5.11 (2.96–8.79)**	***p*** ** ≤ 0.001**
Viral pneumonia	**3.72 (2.35–5.88)**	***p*** ** ≤ 0.001**	**4.09 (2.57–6.49)**	***p*** ** ≤ 0.001**
Bacterial pneumonia	**8.25 (5.76–11.81)**	***p*** ** ≤ 0.001**	**8.09 (5.62–11.64)**	***p*** ** ≤ 0.001**
Other pneumonias due to other or unspecified organisms	**6.52 (4.61–9.23)**	***p*** ** ≤ 0.001**	**6.48 (4.56–9.21)**	***p*** ** ≤ 0.001**
Acute bronchitis	1.03 (0.59–1.8)	0.915	1.14 (0.65–1.99)	0.64
Acute bronchiolitis	1.60 (0.96–2.67)	0.069	**1.75 (1.05–2.92)**	**0.032**
Unspecified acute lower respiratory infection	**3.43 (2.22–5.29)**	***p*** ** ≤ 0.001**	**3.88 (2.51–6.01)**	***p*** ** ≤ 0.001**
Suppurative and necrotic conditions of lower respiratory tract	**23.64 (11.88–47.06)**	***p*** ** ≤ 0.001**	**8.17 (4.05–16.47)**	***p*** ** ≤ 0.001**
COVID-19	**19.12 (13.04–28.03)**	***p*** ** ≤ 0.001**	**7.88 (5.29–11.75)**	***p*** ** ≤ 0.001**
**Year of admission**				
2015	Reference		Reference	
2016	0.95 (0.77–1.17)	0.6	0.90 (0.73–1.12)	0.342
2017	1.04 (0.84–1.27)	0.74	1.00 (0.81–1.23)	0.995
2018	0.88 (0.71–1.09)	0.251	0.82 (0.66–1.02)	0.071
2019	**0.79 (0.63–0.98)**	**0.035**	**0.72 (0.58–0.90)**	**0.004**
2020	**1.96 (1.58–2.42)**	***p*** ** ≤ 0.001**	**1.34 (1.07–1.67)**	**0.01**
2021	**2.79 (2.26–3.45)**	***p*** ** ≤ 0.001**	**2.02 (1.61–2.53)**	***p*** ** ≤ 0.001**
2022	0.92 (0.74–1.14)	0.439	0.90 (0.73–1.12)	0.368
2023	**0.70 (0.57–0.87)**	***p*** ** ≤ 0.001**	**0.69 (0.56–0.86)**	***p*** ** ≤ 0.001**

Note: OR, odds ratio; aOR, adjusted odds ratio; 95% CIs, 95% confidence intervals; N/A, not applicable. Statistically significant effects (*p* < 0.05) are presented in bold. Odds ratios adjusted for age groups, sex, ethnicity, area of residence, healthcare sector, type of acute respiratory infection, and year of hospital admission. Estimates for ethnicity categories such as Black/Afro, Montubio and white should be interpreted cautiously because of sparse events and wide confidence intervals.

**Table 7:** Crude odds ratios and adjusted odds ratios for factors associated with acute respiratory infections in-hospital mortality for the adult sample.

Variables	Adult Population			
	OR (95% CI)	*p*-Value	aOR (95% CI)	*p*-Value
**Age groups**				
0–4 years	N/A		N/A	
5–9 years	N/A		N/A	
10–14 years	N/A		N/A	
15–19 years	N/A		N/A	
20–29 years	Reference		Reference	
30–39 years	**1.44 (1.30–1.61)**	***p*** ** ≤ 0.001**	**1.13 (1.01–1.25)**	**0.034**
40–49 years	**2.55 (2.32–2.82)**	***p*** ** ≤ 0.001**	**1.77 (1.60–1.95)**	***p*** ** ≤ 0.001**
50–59 years	**4.15 (3.78–4.56)**	***p*** ** ≤ 0.001**	**2.84 (2.58–3.12)**	***p*** ** ≤ 0.001**
60–69 years	**6.25 (5.70–6.85)**	***p*** ** ≤ 0.001**	**4.58 (4.18–5.03)**	***p*** ** ≤ 0.001**
≥70 years	**7.37 (6.74–8.06)**	***p*** ** ≤ 0.001**	**7.64 (6.97–8.37)**	***p*** ** ≤ 0.001**
**Sex**				
Male	Reference		Reference	
Female	**0.71 (0.70–0.73)**	***p*** ** ≤ 0.001**	**0.77 (0.75–0.79)**	***p*** ** ≤ 0.001**
**Ethnicity**				
Mestizo	Reference		Reference	
Indigenous	**0.57 (0.52–0.62)**	***p*** ** ≤ 0.001**	**0.69 (0.63–0.75)**	***p*** ** ≤ 0.001**
Black/Afro	0.93 (0.79–1.10)	0.395	1.10 (0.92–1.32)	0.282
Montubio	**0.55 (0.42–0.73)**	***p*** ** ≤ 0.001**	**0.60 (0.45–0.80)**	***p*** ** ≤ 0.001**
White	**0.54 (0.48–0.60)**	***p*** ** ≤ 0.001**	**0.45 (0.40–0.50)**	***p*** ** ≤ 0.001**
Other	**0.92 (0.88–0.97)**	***p*** ** ≤ 0.001**	**0.82 (0.78–0.86)**	***p*** ** ≤ 0.001**
Unknown	**0.94 (0.91–0.97)**	***p*** ** ≤ 0.001**	**0.79 (0.76–0.82)**	***p*** ** ≤ 0.001**
**Area of residence**				
Urban	Reference		Reference	
Rural	**0.94 (0.92–0.97)**	***p*** ** ≤ 0.001**	**0.97 (0.94–0.99)**	**0.04**
**Healthcare sector**				
Public	Reference		Reference	
Private	**0.44 (0.43–0.45)**	***p*** ** ≤ 0.001**	**0.54 (0.52–0.55)**	***p*** ** ≤ 0.001**
**Type of respiratory infections**				
Acute upper respiratory infections	Reference		Reference	
Influenza	**5.32 (3.86–7.32)**	***p*** ** ≤ 0.001**	**4.57 (3.31–6.30)**	***p*** ** ≤ 0.001**
Viral pneumonia	**32.27 (24.69–42.17)**	***p*** ** ≤ 0.001**	**13.83 (10.55–18.13)**	***p*** ** ≤ 0.001**
Bacterial pneumonia	**21.93 (16.90–28.45)**	***p*** ** ≤ 0.001**	**9.23 (7.10–12.00)**	***p*** ** ≤ 0.001**
Other pneumonias due to other or unspecified organisms	**17.41 (13.44–22.57)**	***p*** ** ≤ 0.001**	**7.63 (5.88–9.90)**	***p*** ** ≤ 0.001**
Acute bronchitis	1.09 (0.73–1.62)	0.689	0.68 (0.45–1.02)	0.061
Acute bronchiolitis	**3.67 (1.74–7.75)**	***p*** ** ≤ 0.001**	**2.27 (1.07–4.82)**	**0.033**
Unspecified acute lower respiratory infection	**8.54 (6.31–11.55)**	***p*** ** ≤ 0.001**	**4.28 (3.15–5.80)**	***p*** ** ≤ 0.001**
Suppurative and necrotic conditions of lower respiratory tract	**12.58 (9.17–17.25)**	***p*** ** ≤ 0.001**	**7.50 (5.44–10.32)**	***p*** ** ≤ 0.001**
COVID-19	**39.27 (30.32–50.85)**	***p*** ** ≤ 0.001**	**13.55 (10.43–17.61)**	***p*** ** ≤ 0.001**
**Year of admission**				
2015	Reference		Reference	
2016	1.04 (0.97–1.12)	0.271	**1.10 (1.02–1.18)**	**0.018**
2017	0.96 (0.89–1.03)	0.268	0.95 (0.88–1.03)	0.231
2018	**0.82 (0.76–0.88)**	***p*** ** ≤ 0.001**	**0.85 (0.79–0.92)**	***p*** ** ≤ 0.001**
2019	**0.86 (0.80–0.93)**	***p*** ** ≤ 0.001**	**0.86 (0.79–0.92)**	***p*** ** ≤ 0.001**
2020	**2.39 (2.25–2.53)**	***p*** ** ≤ 0.001**	**1.72 (1.61–1.84)**	***p*** ** ≤ 0.001**
2021	**2.43 (2.30–2.57)**	***p*** ** ≤ 0.001**	**1.76 (1.65–1.89)**	***p*** ** ≤ 0.001**
2022	**1.37 (1.28–1.47)**	***p*** ** ≤ 0.001**	1.02 (0.95–1.09)	0.563
2023	**0.88 (0.82–0.95)**	***p*** ** ≤ 0.001**	**0.83 (0.77–0.89)**	***p*** ** ≤ 0.001**

Note: OR, odds ratio; aOR, adjusted odds ratio; 95% CIs, 95% confidence intervals; N/A, not applicable. Statistically significant effects (*p* < 0.05) are presented in bold. Odds ratios adjusted for age groups, sex, ethnicity, area of residence, healthcare sector, type of acute respiratory infection, and year of hospital admission.

## Data Availability

The original data presented in the study are openly available in the official INEC website (https://www.ecuadorencifras.gob.ec/camas-y-egresos-hospitalarios/, accessed on 1 January 2025). Curated data are available on request from the corresponding author.

## Supplementary Materials

The following supporting information can be downloaded at: https://www.mdpi.com/article/10.3390/medicina62091752/s1, Table S1: Acute respiratory infections hospitalization rates by provinces and by calendar years, per 100,000 inhabitants, for the total study sample; Table S2: Acute respiratory infections in-hospital mortality rates by provinces and by calendar years, per 100,000 inhabitants, for the total study sample; Table S3: Acute respiratory infections hospitalization rates by provinces and by calendar years, per 100,000 inhabitants, for the pediatric sample; Table S4: Acute respiratory infections in-hospital mortality rates by provinces and by calendar years, per 100,000 inhabitants, for the pediatric sample; Table S5: Acute respiratory infections hospitalization rates by provinces and by calendar years, per 100,000 inhabitants, for the adult sample; Table S6: Acute respiratory infections in-hospital mortality rates by provinces and by calendar years, per 100,000 inhabitants, for the adult sample; Table S7: Acute respiratory infections hospitalization rates by type of respiratory infection and by calendar years, per 100,000 inhabitants, for the total study sample; Table S8: Acute respiratory infections in-hospital mortality rates by type of respiratory infection and by calendar years, per 100,000 inhabitants, for the total study sample; Table S9: Acute respiratory infections hospitalization rates by type of respiratory infection and by calendar years, per 100,000 inhabitants, for the pediatric sample; Table S10: Acute respiratory infections in-hospital mortality rates by type of respiratory infection and by calendar years, per 100,000 inhabitants, for the pediatric sample; Table S11: Acute respiratory infections hospitalization rates by type of respiratory infection and by calendar years, per 100,000 inhabitants, for the adult sample; Table S12: Acute respiratory infections in-hospital mortality rates by type of respiratory infection and by calendar years, per 100,000 inhabitants, for the adult sample.
