# Supplementary material for: Acute Respiratory Infections Hospitalizations and In-Hospital Mortality in Ecuador: Differences in Patterns Between Pediatric and Adult Populations
Source: Medicina (Kaunas). 2026 Sep 11;62(9):1752. doi: 10.3390/medicina62091752 (PMC13609215; doi:10.3390/medicina62091752)
Supplement: Supplementary file 1 [file medicina-62-01752-s001.zip › medicina-4539231-supplementary.pdf]

Supplementary table S1. Acute respiratory infections hospitalizations rates by provinces and by calendar years, per 100,000 inhabitants for the total study sample.

Provinces	2015			2016			2017		
	**Cases**	**Population**	**Rates**	**Cases**	**Population**	**Rates**	**Cases**	**Population**	**Rates**
Azuay	3,523	774,638	454.79	3,927	785,615	499.86	3,828	796,212	480.78
Bolívar	380	210,330	180.67	501	210,502	238.00	507	210,337	241.04
Cañar	1,078	235,419	457.91	1,398	236,406	591.36	1,317	237,181	555.27
Carchi	536	175,302	305.76	582	177,819	327.30	687	180,357	380.91
Cotopaxi	1,314	443,863	296.04	1,337	450,184	296.99	1,295	456,171	283.88
Chimborazo	1,978	495,036	399.57	2,603	497,820	522.88	2,414	499,658	483.13
El Oro	2,811	683,445	411.30	3,508	695,673	504.26	3,031	707,579	428.36
Esmeraldas	841	567,967	148.07	897	573,816	156.32	1,055	579,132	182.17
Guayas	10,760	4,158,440	258.75	11,442	4,235,294	270.16	9,882	4,315,276	229.00
Imbabura	1,424	451,794	315.19	1,568	457,639	342.63	1,837	463,284	396.52
Loja	1,638	494,669	331.13	1,744	496,260	351.43	1,952	497,317	392.51
Los Ríos	1,808	867,790	208.35	2,091	883,393	236.70	2,267	899,364	252.07
Manabí	3,524	1,527,909	230.64	3,824	1,552,094	246.38	4,076	1,576,190	258.60
Morona Santiago	1,378	174,078	791.60	1,645	177,950	924.42	1,827	181,784	1005.04
Napo	373	119,718	311.57	415	122,096	339.90	361	124,453	290.07
Pastaza	431	98,370	438.14	519	101,047	513.62	430	103,642	414.89
Pichincha	8,575	2,931,254	292.54	11,738	2,987,359	392.92	11,948	3,043,523	392.57
Tungurahua	1,986	546,729	363.25	2,368	552,241	428.80	2,249	557,286	403.56
Zamora Chimchipe	299	104,161	287.06	340	105,922	320.99	415	107,662	385.47
Galápagos	54	27,284	197.92	77	27,579	279.20	86	27,883	308.43
Sucumbíos	881	196,773	447.72	734	199,696	367.56	629	202,373	310.81
Orellana	286	159,355	179.47	388	163,582	237.19	367	167,616	218.95

Santo Domingo de los Tsáchilas	1,382	469,484	294.37	1,502	478,160	314.12	1,307	485,919	268.97
Santa Elena	765	355,363	215.27	936	364,118	257.06	1,051	372,604	282.07
Total	48,025	16,269,171	295.19	56,084	16,532,265	339.24	54,818	16,792,803	326.44

Supplementary table S1. (Continued**)**

**Provinces**	**2018**			**2019**			**2020**		
	**Cases**	**Population**	**Rates**	**Cases**	**Population**	**Rates**	**Cases**	**Population**	**Rates**
Azuay	4,364	808,420	539.82	3,896	820,239	474.98	6,001	825,590	726.87
Bolívar	550	209,822	262.13	530	209,046	253.53	863	207,926	415.05
Cañar	1,704	237,689	716.90	1,444	237,967	606.81	1,463	238,020	614.65
Carchi	673	182,430	368.91	650	183,616	354.00	789	183,603	429.73
Cotopaxi	1,407	461,715	304.73	1,229	466,924	263.21	1,920	471,628	407.10
Chimborazo	2,304	500,340	460.49	2,126	499,939	425.25	2,845	498,371	570.86
El Oro	2,730	718,677	379.86	2,374	728,281	325.97	3,837	734,775	522.20
Esmeraldas	1,511	583,774	258.83	1,304	587,721	221.87	1,349	590,909	228.29
Guayas	9,979	4,410,700	226.25	11,936	4,509,756	264.67	15,142	4,568,499	331.44
Imbabura	1,943	468,835	414.43	1,663	474,152	350.73	2,234	478,631	466.75
Loja	2,269	497,863	455.75	2,030	497,948	407.67	2,560	497,465	514.61
Los Ríos	2,292	917,992	249.68	1,869	936,316	199.61	2,595	945,760	274.38
Manabí	4,366	1,604,308	272.14	3,958	1,633,131	242.36	4,745	1,650,334	287.52
Morona Santiago	2,074	185,624	1117.31	1,651	189,402	871.69	1,470	192,900	762.05
Napo	329	126,836	259.39	387	129,165	299.62	498	131,206	379.56
Pastaza	436	105,998	411.33	473	108,179	437.24	432	110,462	391.08

Pichincha	14,218	3,107,396	457.55	12,991	3,170,064	409.80	27,040	3,204,953	843.69
Tungurahua	2,806	562,196	499.11	2,686	566,737	473.94	4,792	569,872	840.89
Zamora Chimchipe	567	109,489	517.86	491	111,300	441.15	476	112,755	422.15
Galápagos	87	28,205	308.46	80	28,530	280.41	90	28,842	312.04
Sucumbíos	696	204,827	339.80	630	206,756	304.71	933	207,715	449.17
Orellana	260	171,519	151.59	243	175,338	138.59	434	179,067	242.37
Santo Domingo de los Tsáchilas	1,631	492,964	330.86	1,345	499,418	269.31	2,686	504,662	532.24
Santa Elena	1,053	380,264	276.91	912	386,955	235.69	1,192	392,510	303.69
Total	60,249	17,077,883	352.79	56,898	17,356,880	327.81	86,386	17,526,455	492.89

Supplementary table S1. (Continued)

**Provinces**	**2021**			**2022**			**2023**			**Average rates 2015-2023**
	**Cases**	**Population**	**Rates**	**Cases**	**Population**	**Rates**	**Cases**	**Population**	**Rates**	
Azuay	5,547	825,773	671.73	4,750	826,246	574.89	5,067	828,172	611.83	559.51
Bolívar	911	206,608	440.93	624	205,544	303.58	797	204,819	389.12	302.67
Cañar	1,384	237,790	582.03	1,544	237,584	649.88	1,477	237,527	621.82	599.62
Carchi	991	182,445	543.18	621	180,776	343.52	549	179,142	306.46	373.31
Cotopaxi	1,830	475,870	384.56	1,510	480,270	314.41	1,518	484,792	313.12	318.23
Chimborazo	2,637	495,781	531.89	2,273	493,084	460.98	2,308	490,774	470.28	480.59
El Oro	4,189	738,399	567.31	2,866	741,473	386.53	2,364	744,811	317.40	427.02
Esmeraldas	1,154	593,324	194.50	993	595,674	166.70	1,018	598,430	170.11	191.87
Guayas	15,354	4,601,037	333.71	11,264	4,643,432	242.58	10,880	4,690,613	231.95	265.39

Imbabura	2,681	482,224	555.97	2,045	485,913	420.86	2,069	489,961	422.28	409.48
Loja	3,204	496,526	645.28	2,606	496,194	525.20	2,661	496,685	535.75	462.15
Los Ríos	2,588	948,792	272.77	2,181	953,060	228.84	1,985	959,805	206.81	236.58
Manabí	4,711	1,659,426	283.89	3,894	1,670,700	233.08	4,015	1,684,317	238.38	254.78
Morona Santiago	1,333	196,157	679.56	1,367	199,453	685.37	1,530	202,851	754.25	843.48
Napo	438	132,987	329.36	343	134,768	254.51	410	136,622	300.10	307.12
Pastaza	562	112,771	498.36	426	114,969	370.53	456	117,062	389.54	429.41
Pichincha	25,116	3,217,582	780.59	17,530	3,231,547	542.46	19,767	3,250,537	608.11	524.47
Tungurahua	3,812	571,693	666.79	2,933	573,786	511.17	3,314	576,407	574.94	529.16
Zamora Chinchipe	440	113,912	386.26	563	115,101	489.14	669	116,363	574.93	425.00
Galápagos	113	29,148	387.68	71	29,463	240.98	71	29,808	238.19	283.70
Sucumbíos	798	207,728	384.16	488	207,221	235.50	491	206,360	237.93	341.93
Orellana	458	182,673	250.72	324	186,166	174.04	344	189,568	181.47	197.15
Santo Domingo de los Tsáchilas	2,431	509,041	477.56	1,951	513,692	379.80	1,627	518,576	313.74	353.44
Santa Elena	757	396,709	190.82	859	399,185	215.19	883	400,829	220.29	244.11
Total	83,439	17,614,396	473.70	64,026	17,715,301	361.42	66,270	17,834,831	371.58	371.23

Supplementary table S2. Acute respiratory infections in-hospital mortality rates by provinces and by calendar years, per 100,000 inhabitants, for the total study sample.

Provinces	2015			2016			2017		
	Cases	Population	Rates	Cases	Population	Rates	Cases	Population	Rates
Azuay	140	774,638	18.07	180	785,615	22.91	205	796,212	25.75
Bolívar	13	210,330	6.18	18	210,502	8.55	14	210,337	6.66
Cañar	27	235,419	11.47	34	236,406	14.38	42	237,181	17.71
Carchi	29	175,302	16.54	23	177,819	12.93	16	180,357	8.87
Cotopaxi	43	443,863	9.69	29	450,184	6.44	27	456,171	5.92
Chimborazo	44	495,036	8.89	72	497,820	14.46	70	499,658	14.01
El Oro	135	683,445	19.75	132	695,673	18.97	140	707,579	19.79
Esmeraldas	28	567,967	4.93	31	573,816	5.40	35	579,132	6.04
Guayas	454	4,158,440	10.92	588	4,235,294	13.88	504	4,315,276	11.68
Imbabura	23	451,794	5.09	36	457,639	7.87	41	463,284	8.85
Loja	64	494,669	12.94	60	496,260	12.09	48	497,317	9.65
Los Ríos	51	867,790	5.88	44	883,393	4.98	46	899,364	5.11
Manabí	71	1,527,909	4.65	144	1,552,094	9.28	93	1,576,190	5.90
Morona Santiago	25	174,078	14.36	22	177,950	12.36	32	181,784	17.60
Napo	8	119,718	6.68	6	122,096	4.91	3	124,453	2.41
Pastaza	0	98,370	0.00	9	101,047	8.91	8	103,642	7.72
Pichincha	264	2,931,254	9.01	319	2,987,359	10.68	318	3,043,523	10.45
Tungurahua	111	546,729	20.30	82	552,241	14.85	83	557,286	14.89
Zamora Chinchipe	8	104,161	7.68	9	105,922	8.50	9	107,662	8.36
Galápagos	3	27,284	11.00	2	27,579	7.25	0	27,883	0.00
Sucumbíos	12	196,773	6.10	9	199,696	4.51	15	202,373	7.41
Orellana	6	159,355	3.77	2	163,582	1.22	4	167,616	2.39

Santo Domingo de los Tsáchilas	26	469,484	5.54	34	478,160	7.11	22	485,919	4.53
Santa Elena	18	355,363	5.07	27	364,118	7.42	17	372,604	4.56
Total	1,603	16,269,171	9.85	1,912	16,532,265	11.57	1,792	16,792,803	10.67

Supplementary table S2. (Continued**)**

**Provinces**	**2018**			**2019**			**2020**		
	**Cases**	**Population**	**Rates**	**Cases**	**Population**	**Rates**	**Cases**	**Population**	**Rates**
Azuay	176	808,420	21.77	163	820,239	19.87	957	825,590	115.92
Bolívar	8	209,822	3.81	13	209,046	6.22	88	207,926	42.32
Cañar	53	237,689	22.30	38	237,967	15.97	265	238,020	111.34
Carchi	26	182,430	14.25	27	183,616	14.70	157	183,603	85.51
Cotopaxi	42	461,715	9.10	32	466,924	6.85	165	471,628	34.99
Chimborazo	59	500,340	11.79	67	499,939	13.40	569	498,371	114.17
El Oro	86	718,677	11.97	80	728,281	10.98	1,181	734,775	160.73
Esmeraldas	29	583,774	4.97	30	587,721	5.10	286	590,909	48.40
Guayas	407	4,410,700	9.23	468	4,509,756	10.38	2,373	4,568,499	51.94
Imbabura	42	468,835	8.96	27	474,152	5.69	359	478,631	75.01
Loja	55	497,863	11.05	52	497,948	10.44	370	497,465	74.38
Los Ríos	53	917,992	5.77	28	936,316	2.99	301	945,760	31.83
Manabí	95	1,604,308	5.92	103	1,633,131	6.31	981	1,650,334	59.44
Morona Santiago	31	185,624	16.70	20	189,402	10.56	81	192,900	41.99
Napo	4	126,836	3.15	11	129,165	8.52	91	131,206	69.36
Pastaza	11	105,998	10.38	13	108,179	12.02	58	110,462	52.51

Pichincha	406	3,107,396	13.07	341	3,170,064	10.76	3,031	3,204,953	94.57
Tungurahua	115	562,196	20.46	95	566,737	16.76	926	569,872	162.49
Zamora Chinchipe	13	109,489	11.87	5	111,300	4.49	56	112,755	49.67
Galápagos	0	28,205	0.00	1	28,530	3.51	14	28,842	48.54
Sucumbíos	31	204,827	15.13	15	206,756	7.25	153	207,715	73.66
Orellana	2	171,519	1.17	5	175,338	2.85	60	179,067	33.51
Santo Domingo de los Tsáchilas	40	492,964	8.11	55	499,418	11.01	528	504,662	104.62
Santa Elena	24	380,264	6.31	13	386,955	3.36	211	392,510	53.76
Total	1,808	17,077,883	10.59	1,702	17,356,880	9.81	13,261	17,526,455	75.66

Supplementary table S2. (Continued**)**

**Provinces**	**2021**			**2022**			**2023**			**Average rates 2015-2023**
	**Cases**	**Population**	**Rates**	**Cases**	**Population**	**Rates**	**Cases**	**Population**	**Rates**	
Azuay	1,077	825,773	130.42	341	826,246	41.27	192	828,172	23.18	46.57
Bolívar	89	206,608	43.08	13	205,544	6.32	11	204,819	5.37	14.28
Cañar	206	237,790	86.63	53	237,584	22.31	37	237,527	15.58	35.30
Carchi	197	182,445	107.98	20	180,776	11.06	17	179,142	9.49	31.26
Cotopaxi	169	475,870	35.51	58	480,270	12.08	40	484,792	8.25	14.31
Chimborazo	504	495,781	101.66	107	493,084	21.70	43	490,774	8.76	34.32
El Oro	1,378	738,399	186.62	306	741,473	41.27	92	744,811	12.35	53.60
Esmeraldas	220	593,324	37.08	34	595,674	5.71	33	598,430	5.51	13.68
Guayas	1,951	4,601,037	42.40	589	4,643,432	12.68	389	4,690,613	8.29	19.05

Imbabura	446	482,224	92.49	75	485,913	15.43	34	489,961	6.94	25.15
Loja	639	496,526	128.69	146	496,194	29.42	73	496,685	14.70	33.71
Los Ríos	300	948,792	31.62	107	953,060	11.23	74	959,805	7.71	11.90
Manabí	1,136	1,659,426	68.46	295	1,670,700	17.66	141	1,684,317	8.37	20.66
Morona Santiago	72	196,157	36.71	33	199,453	16.55	21	202,851	10.35	19.69
Napo	71	132,987	53.39	15	134,768	11.13	6	136,622	4.39	18.22
Pastaza	82	112,771	72.71	13	114,969	11.31	4	117,062	3.42	19.89
Pichincha	3,415	3,217,582	106.14	763	3,231,547	23.61	309	3,250,537	9.51	31.98
Tungurahua	876	571,693	153.23	122	573,786	21.26	54	576,407	9.37	48.18
Zamora Chinchipe	67	113,912	58.82	20	115,101	17.38	16	116,363	13.75	20.06
Galápagos	16	29,148	54.89	1	29,463	3.39	3	29,808	10.06	15.40
Sucumbíos	178	207,728	85.69	40	207,221	19.30	21	206,360	10.18	25.47
Orellana	56	182,673	30.66	10	186,166	5.37	6	189,568	3.17	9.34
Santo Domingo de los Tsáchilas	596	509,041	117.08	160	513,692	31.15	81	518,576	15.62	33.86
Santa Elena	161	396,709	40.58	39	399,185	9.77	11	400,829	2.74	14.84
Total	13,902	17,614,396	78.92	3,360	17,715,301	18.97	1,708	17,834,831	9.58	26.18

Supplementary table S3. Acute respiratory infections hospitalizations rates by provinces and by calendar years, per 100,000 inhabitants for the pediatric sample.

**Provinces**	**2015**			**2016**			**2017**		
	**Cases**	**Population**	**Rates**	**Cases**	**Population**	**Rates**	**Cases**	**Population**	**Rates**
Azuay	1,999	290,197	688.84	2,116	289,844	730.05	2,066	289,472	713.71
Bolívar	208	87,238	238.43	297	85,835	346.01	297	84,253	352.51
Cañar	591	98,058	602.70	766	97,467	785.91	727	96,805	750.99
Carchi	283	64,510	438.69	349	64,368	542.19	386	64,211	601.14
Cotopaxi	830	182,033	455.96	821	181,298	452.85	816	180,238	452.73
Chimborazo	1,330	194,057	685.37	1,689	191,361	882.62	1,503	188,313	798.14
El Oro	1,896	256,698	738.61	2,372	256,713	923.99	1,910	256,510	744.61
Esmeraldas	576	254,340	226.47	630	253,290	248.73	697	251,863	276.74
Guayas	8,003	1,629,534	491.12	8,219	1,639,481	501.32	6,963	1,648,147	422.47
Imbabura	1,018	180,091	565.27	1,078	179,213	601.52	1,236	178,079	694.07
Loja	800	192,183	416.27	914	189,928	481.23	1,169	187,499	623.47
Los Ríos	1,183	348,922	339.04	1,384	350,222	395.18	1,495	351,413	425.43
Manabí	2,488	611,042	407.17	2,549	609,499	418.21	2,959	607,780	486.85
Morona Santiago	998	90,806	1099.05	1,210	91,408	1323.74	1,315	91,880	1431.21
Napo	234	57,042	410.22	327	57,398	569.71	297	57,698	514.75
Pastaza	324	45,807	707.32	446	46,593	957.23	376	47,284	795.19
Pichincha	5,445	1,012,512	537.77	7,903	1,015,103	778.54	7,821	1,016,279	769.57
Tungurahua	1,203	203,780	590.34	1,647	202,779	812.21	1,494	201,540	741.29
Zamora Chinchipe	215	48,471	443.56	236	48,344	488.17	273	48,160	566.86
Galápagos	26	9,409	276.33	46	9,425	488.06	56	9,432	593.72
Sucumbíos	650	89,470	726.50	595	89,593	664.11	487	89,508	544.09
Orellana	227	75,652	300.06	314	76,891	408.37	286	77,929	367.00

Santo Domingo de los Tsáchilas	959	195,962	489.38	944	197,545	477.87	876	198,487	441.34
Santa Elena	500	142,103	351.86	644	143,984	447.27	779	145,651	534.84
Total	31,986	6,359,917	502.93	37,496	6,367,582	588.86	36,284	6,368,431	569.75

Supplementary table S3. (Continued**)**

**Provinces**	**2018**			**2019**			**2020**		
	**Cases**	**Population**	**Rates**	**Cases**	**Population**	**Rates**	**Cases**	**Population**	**Rates**
Azuay	2,098	289,490	724.72	2,270	289,553	783.97	1,237	288,630	428.58
Bolívar	308	82,506	373.31	365	80,642	452.62	170	78,712	215.98
Cañar	863	96,068	898.32	785	95,261	824.05	414	94,393	438.59
Carchi	374	63,920	585.11	390	63,394	615.20	177	62,598	282.76
Cotopaxi	797	178,851	445.62	734	177,206	414.21	483	175,380	275.40
Chimborazo	1,306	184,911	706.29	1,222	181,203	674.38	582	177,270	328.31
El Oro	1,696	256,082	662.29	1,551	255,405	607.27	544	254,400	213.84
Esmeraldas	1,021	250,047	408.32	911	247,865	367.54	429	245,378	174.83
Guayas	7,046	1,658,056	424.96	8,371	1,666,574	502.29	3,347	1,667,071	200.77
Imbabura	1,216	176,736	688.03	1,066	175,190	608.48	534	173,376	308.00
Loja	1,084	184,955	586.09	1,022	182,351	560.46	474	179,838	263.57
Los Ríos	1,540	352,937	436.34	1,226	354,215	346.12	525	353,884	148.35
Manabí	3,150	606,718	519.19	2,717	605,469	448.74	900	601,917	149.52
Morona Santiago	1,538	92,247	1667.26	1,165	92,502	1259.43	713	92,631	769.72
Napo	272	57,949	469.38	290	58,139	498.80	168	58,247	288.43
Pastaza	372	47,835	777.67	388	48,256	804.05	189	48,627	388.67

Pichincha	8,962	1,016,920	881.29	8,385	1,015,116	826.01	4,652	1,006,922	462.00
Tungurahua	1,652	200,143	825.41	1,659	198,621	835.26	837	196,943	425.00
Zamora Chinchipe	369	47,959	769.41	348	47,726	729.16	172	47,409	362.80
Galápagos	55	9,429	583.31	45	9,394	479.03	31	9,357	331.30
Sucumbíos	479	89,214	536.91	470	88,592	530.52	234	87,558	267.25
Orellana	195	78,787	247.50	192	79,496	241.52	127	80,116	158.52
Santo Domingo de los Tsáchilas	1,006	198,966	505.61	846	199,069	424.98	463	198,828	232.86
Santa Elena	780	146,892	531.00	656	147,676	444.22	196	148,144	132.30
Total	38,179	6,367,618	599.58	37,074	6,358,915	583.02	17,598	6,327,629	278.11

Supplementary table S3. (Continued**)**

Provinces	2021			2022			2023			Average rates 2015-2023
	Cases	Population	Rates	Cases	Population	Rates	Cases	Population	Rates	
Azuay	483	287,008	168.29	2,213	285,405	775.39	3,030	283,851	1067.46	675.67
Bolívar	121	76,756	157.64	428	74,832	571.95	643	73,011	880.69	398.79
Cañar	191	93,437	204.42	743	92,382	804.27	837	91,242	917.34	691.84
Carchi	94	61,549	152.72	423	60,360	700.80	376	59,160	635.56	506.02
Cotopaxi	164	173,366	94.60	888	171,151	518.84	1,022	168,769	605.56	412.86
Chimborazo	278	173,188	160.52	1,271	169,064	751.79	1,621	165,109	981.78	663.24
El Oro	611	253,014	241.49	1,545	251,337	614.71	1,648	249,542	660.41	600.80
Esmeraldas	390	242,593	160.76	651	239,662	271.63	661	236,815	279.12	268.24
Guayas	3,372	1,661,247	202.98	7,179	1,653,783	434.10	7,971	1,645,223	484.49	407.17

Imbabura	298	171,328	173.94	1,127	169,186	666.13	1,394	167,016	834.65	571.12
Loja	306	177,430	172.46	1,265	175,117	722.37	1,574	172,902	910.34	526.25
Los Ríos	610	352,272	173.16	1,349	350,388	385.00	1,439	348,573	412.83	340.16
Manabí	964	596,670	161.56	2,367	591,352	400.27	3,006	586,285	512.72	389.36
Morona Santiago	633	92,654	683.19	939	92,616	1013.86	1,093	92,532	1181.21	1,158.74
Napo	90	58,268	154.46	236	58,204	405.47	348	58,081	599.16	434.49
Pastaza	204	48,918	417.02	314	49,087	639.68	387	49,153	787.34	697.13
Pichincha	2,223	993,997	223.64	9,986	979,934	1019.05	14,613	965,563	1513.42	779.03
Tungurahua	320	195,054	164.06	1,768	192,966	916.22	2,332	190,692	1222.91	725.86
Zamora Chinchipe	128	47,020	272.22	360	46,603	772.48	526	46,169	1139.29	616.00
Galápagos	20	9,333	214.29	49	9,300	526.88	46	9,256	496.97	443.32
Sucumbíos	177	86,161	205.43	287	84,500	339.64	354	82,673	428.19	471.41
Orellana	106	80,613	131.49	173	80,938	213.74	235	81,109	289.73	261.99
Santo Domingo de los Tsáchilas	464	198,273	234.02	1,123	197,459	568.73	1,133	196,495	576.61	439.04
Santa Elena	84	148,132	56.71	593	147,189	402.88	711	145,758	487.79	376.54
Total	12,331	6,278,281	196.41	37,277	6,222,815	599.04	47,000	6,164,979	762.37	520.01

Supplementary table S4. Acute respiratory infections in-hospital mortality rates by provinces and by calendar years, per 100,000 inhabitants for the pediatric sample.

Provinces	2015			2016			2017		
	Cases	Population	Rates	Cases	Population	Rates	Cases	Population	Rates
Azuay	11	290,197	3.79	14	289,844	4.83	22	289,472	7.60
Bolívar	1	87,238	1.15	3	85,835	3.50	1	84,253	1.19
Cañar	1	98,058	1.02	0	97,467	0.00	0	96,805	0.00
Carchi	1	64,510	1.55	0	64,368	0.00	2	64,211	3.11
Cotopaxi	10	182,033	5.49	4	181,298	2.21	4	180,238	2.22
Chimborazo	3	194,057	1.55	4	191,361	2.09	4	188,313	2.12
El Oro	13	256,698	5.06	12	256,713	4.67	8	256,510	3.12
Esmeraldas	6	254,340	2.36	5	253,290	1.97	7	251,863	2.78
Guayas	45	1,629,534	2.76	40	1,639,481	2.44	47	1,648,147	2.85
Imbabura	3	180,091	1.67	2	179,213	1.12	0	178,079	0.00
Loja	3	192,183	1.56	3	189,928	1.58	2	187,499	1.07
Los Ríos	4	348,922	1.15	5	350,222	1.43	4	351,413	1.14
Manabí	6	611,042	0.98	15	609,499	2.46	8	607,780	1.32
Morona Santiago	10	90,806	11.01	7	91,408	7.66	13	91,880	14.15
Napo	2	57,042	3.51	2	57,398	3.48	0	57,698	0.00
Pastaza	0	45,807	0.00	2	46,593	4.29	4	47,284	8.46
Pichincha	29	1,012,512	2.86	51	1,015,103	5.02	58	1,016,279	5.71
Tungurahua	7	203,780	3.44	6	202,779	2.96	4	201,540	1.98
Zamora Chinchipe	1	48,471	2.06	1	48,344	2.07	1	48,160	2.08
Galápagos	0	9,409	0.00	0	9,425	0.00	0	9,432	0.00
Sucumbíos	4	89,470	4.47	4	89,593	4.46	3	89,508	3.35
Orellana	2	75,652	2.64	1	76,891	1.30	1	77,929	1.28

Santo Domingo de los Tsáchilas	3	195,962	1.53	3	197,545	1.52	1	198,487	0.50
Santa Elena	1	142,103	0.70	0	143,984	0.00	1	145,651	0.69
Total	166	6,359,917	2.61	184	6,367,582	2.89	195	6,368,431	3.06

Supplementary table S4. (Continued**)**

**Provinces**	**2018**			**2019**			**2020**		
	**Cases**	**Population**	**Rates**	**Cases**	**Population**	**Rates**	**Cases**	**Population**	**Rates**
Azuay	14	289,490	4.84	15	289,553	5.18	12	288,630	4.16
Bolívar	0	82,506	0.00	0	80,642	0.00	2	78,712	2.54
Cañar	1	96,068	1.04	1	95,261	1.05	3	94,393	3.18
Carchi	1	63,920	1.56	1	63,394	1.58	1	62,598	1.60
Cotopaxi	1	178,851	0.56	3	177,206	1.69	1	175,380	0.57
Chimborazo	2	184,911	1.08	3	181,203	1.66	3	177,270	1.69
El Oro	0	256,082	0.00	2	255,405	0.78	6	254,400	2.36
Esmeraldas	9	250,047	3.60	5	247,865	2.02	12	245,378	4.89
Guayas	38	1,658,056	2.29	40	1,666,574	2.40	35	1,667,071	2.10
Imbabura	1	176,736	0.57	0	175,190	0.00	2	173,376	1.15
Loja	2	184,955	1.08	1	182,351	0.55	5	179,838	2.78
Los Ríos	3	352,937	0.85	3	354,215	0.85	3	353,884	0.85
Manabí	9	606,718	1.48	10	605,469	1.65	14	601,917	2.33
Morona Santiago	7	92,247	7.59	7	92,502	7.57	2	92,631	2.16
Napo	1	57,949	1.73	0	58,139	0.00	6	58,247	10.30
Pastaza	1	47,835	2.09	0	48,256	0.00	2	48,627	4.11

Pichincha	68	1,016,920	6.69	47	1,015,116	4.63	56	1,006,922	5.56
Tungurahua	6	200,143	3.00	4	198,621	2.01	5	196,943	2.54
Zamora Chinchipe	0	47,959	0.00	0	47,726	0.00	1	47,409	2.11
Galápagos	0	9,429	0.00	0	9,394	0.00	0	9,357	0.00
Sucumbíos	7	89,214	7.85	3	88,592	3.39	1	87,558	1.14
Orellana	0	78,787	0.00	0	79,496	0.00	1	80,116	1.25
Santo Domingo de los Tsáchilas	4	198,966	2.01	4	199,069	2.01	5	198,828	2.51
Santa Elena	0	146,892	0.00	2	147,676	1.35	0	148,144	0.00
Total	175	6,367,618	2.75	151	6,358,915	2.37	178	6,327,629	2.81

Supplementary table S4. (Continued**)**

**Provinces**	**2021**			**2022**			**2023**			**Average rates 2015-2023**
	**Cases**	**Population**	**Rates**	**Cases**	**Population**	**Rates**	**Cases**	**Population**	**Rates**	
Azuay	15	287,008	5.23	28	285,405	9.81	22	283,851	7.75	5.91
Bolívar	0	76,756	0.00	0	74,832	0.00	1	73,011	1.37	1.08
Cañar	1	93,437	1.07	2	92,382	2.16	2	91,242	2.19	1.30
Carchi	1	61,549	1.62	4	60,360	6.63	1	59,160	1.69	2.15
Cotopaxi	1	173,366	0.58	4	171,151	2.34	4	168,769	2.37	2.00
Chimborazo	1	173,188	0.58	4	169,064	2.37	1	165,109	0.61	1.53
El Oro	8	253,014	3.16	11	251,337	4.38	3	249,542	1.20	2.75
Esmeraldas	1	242,593	0.41	2	239,662	0.83	6	236,815	2.53	2.38
Guayas	41	1,661,247	2.47	38	1,653,783	2.30	51	1,645,223	3.10	2.52

Imbabura	4	171,328	2.33	3	169,186	1.77	5	167,016	2.99	1.29
Loja	1	177,430	0.56	3	175,117	1.71	3	172,902	1.74	1.40
Los Ríos	2	352,272	0.57	6	350,388	1.71	2	348,573	0.57	1.01
Manabí	42	596,670	7.04	11	591,352	1.86	16	586,285	2.73	2.43
Morona Santiago	5	92,654	5.40	4	92,616	4.32	7	92,532	7.56	7.49
Napo	1	58,268	1.72	2	58,204	3.44	0	58,081	0.00	2.69
Pastaza	1	48,918	2.04	3	49,087	6.11	0	49,153	0.00	3.01
Pichincha	45	993,997	4.53	39	979,934	3.98	29	965,563	3.00	4.66
Tungurahua	1	195,054	0.51	3	192,966	1.55	3	190,692	1.57	2.17
Zamora Chinchipe	0	47,020	0.00	0	46,603	0.00	1	46,169	2.17	1.16
Galápagos	0	9,333	0.00	0	9,300	0.00	0	9,256	0.00	0.00
Sucumbíos	1	86,161	1.16	3	84,500	3.55	3	82,673	3.63	3.67
Orellana	1	80,613	1.24	1	80,938	1.24	2	81,109	2.47	1.27
Santo Domingo de los Tsáchilas	4	198,273	2.02	7	197,459	3.55	9	196,495	4.58	2.25
Santa Elena	0	148,132	0.00	0	147,189	0.00	0	145,758	0.00	0.30
Total	177	6,278,281	2.82	178	6,222,815	2.86	171	6,164,979	2.77	2.77

Supplementary table S5. Acute respiratory infections hospitalizations rates by provinces and by calendar years, per 100,000 inhabitants for the adult sample.

Provinces	2015			2016			2017		
	Cases	Population	Rates	Cases	Population	Rates	Cases	Population	Rates
Azuay	1,524	484,441	314.59	1,811	495,771	365.29	1,762	506,740	347.71
Bolívar	172	123,092	139.73	204	124,667	163.64	210	126,084	166.56
Cañar	487	137,361	354.54	632	138,939	454.88	590	140,376	420.30
Carchi	253	110,792	228.36	233	113,451	205.38	301	116,146	259.16
Cotopaxi	484	261,830	184.85	516	268,886	191.90	479	275,933	173.59
Chimborazo	648	300,979	215.30	914	306,459	298.25	911	311,345	292.60
El Oro	915	426,747	214.41	1,136	438,960	258.79	1,121	451,069	248.52
Esmeraldas	265	313,627	84.50	267	320,526	83.30	358	327,269	109.39
Guayas	2,757	2,528,906	109.02	3,223	2,595,813	124.16	2,919	2,667,129	109.44
Imbabura	406	271,703	149.43	490	278,426	175.99	601	285,205	210.73
Loja	838	302,486	277.04	830	306,332	270.95	783	309,818	252.73
Los Ríos	625	518,868	120.45	707	533,171	132.60	772	547,951	140.89
Manabí	1,036	916,867	112.99	1,275	942,595	135.26	1,117	968,410	115.34
Morona Santiago	380	83,272	456.34	435	86,542	502.65	512	89,904	569.50
Napo	139	62,676	221.78	88	64,698	136.02	64	66,755	95.87
Pastaza	107	52,563	203.57	73	54,454	134.06	54	56,358	95.82
Pichincha	3,130	1,918,742	163.13	3,835	1,972,256	194.45	4,127	2,027,244	203.58
Tungurahua	783	342,949	228.31	721	349,462	206.32	755	355,746	212.23
Zamora Chinchipe	84	55,690	150.83	104	57,578	180.62	142	59,502	238.65
Galápagos	28	17,875	156.64	31	18,154	170.76	30	18,451	162.59
Sucumbíos	231	107,303	215.28	139	110,103	126.25	142	112,865	125.81
Orellana	59	83,703	70.49	74	86,691	85.36	81	89,687	90.31

Santo Domingo de los Tsáchilas	423	273,522	154.65	558	280,615	198.85	431	287,432	149.95
Santa Elena	265	213,260	124.26	292	220,134	132.65	272	226,953	119.85
Total	16,039	9,909,254	161.86	18,588	10,164,683	182.87	18,534	10,424,372	177.79

Supplementary table S5. (Continued**)**

**Provinces**	**2018**			**2019**			**2020**		
	**Cases**	**Population**	**Rates**	**Cases**	**Population**	**Rates**	**Cases**	**Population**	**Rates**
Azuay	2,266	518,930	436.67	1,626	530,686	306.40	4,764	536,960	887.22
Bolívar	242	127,316	190.08	165	128,404	128.50	693	129,214	536.32
Cañar	841	141,621	593.84	659	142,706	461.79	1,049	143,627	730.36
Carchi	299	118,510	252.30	260	120,222	216.27	612	121,005	505.76
Cotopaxi	610	282,864	215.65	495	289,718	170.86	1,437	296,248	485.07
Chimborazo	998	315,429	316.39	904	318,736	283.62	2,263	321,101	704.76
El Oro	1,034	462,595	223.52	823	472,876	174.04	3,293	480,375	685.51
Esmeraldas	490	333,727	146.83	393	339,856	115.64	920	345,531	266.26
Guayas	2,933	2,752,644	106.55	3,565	2,843,182	125.39	11,795	2,901,428	406.52
Imbabura	727	292,099	248.89	597	298,962	199.69	1,700	305,255	556.91
Loja	1,185	312,908	378.71	1,008	315,597	319.39	2,086	317,627	656.75
Los Ríos	752	565,055	133.08	643	582,101	110.46	2,070	591,876	349.74
Manabí	1,216	997,590	121.89	1,241	1,027,662	120.76	3,845	1,048,417	366.74
Morona Santiago	536	93,377	574.02	486	96,900	501.55	757	100,269	754.97
Napo	57	68,887	82.74	97	71,026	136.57	330	72,959	452.31
Pastaza	64	58,163	110.04	85	59,923	141.85	243	61,835	392.98

Pichincha	5,256	2,090,476	251.43	4,606	2,154,948	213.74	22,388	2,198,031	1018.55
Tungurahua	1,154	362,053	318.74	1,027	368,116	278.99	3,955	372,929	1060.52
Zamora Chinchipe	198	61,530	321.79	143	63,574	224.93	304	65,346	465.22
Galápagos	32	18,776	170.43	35	19,136	182.90	59	19,485	302.80
Sucumbíos	217	115,613	187.70	160	118,164	135.41	699	120,157	581.74
Orellana	65	92,732	70.09	51	95,842	53.21	307	98,951	310.25
Santo Domingo de los Tsáchilas	625	293,998	212.59	499	300,349	166.14	2,223	305,834	726.86
Santa Elena	273	233,372	116.98	256	239,279	106.99	996	244,366	407.59
Total	22,070	10,710,265	206.06	19,824	10,997,965	180.25	68,788	11,198,826	614.24

Supplementary table S5. (Continued**)**

**Provinces**	**2021**			**2022**			**2023**			**Average rates 2015-2023**
	**Cases**	**Population**	**Rates**	**Cases**	**Population**	**Rates**	**Cases**	**Population**	**Rates**	
Azuay	5,064	538,765	939.93	2,537	540,841	469.08	2,037	544,321	374.23	493.46
Bolívar	790	129,852	608.38	196	130,712	149.95	154	131,808	116.84	244.44
Cañar	1,193	144,353	826.45	801	145,202	551.65	640	146,285	437.50	536.81
Carchi	897	120,896	741.96	198	120,416	164.43	173	119,982	144.19	301.98
Cotopaxi	1,666	302,504	550.74	622	309,119	201.22	496	316,023	156.95	258.98
Chimborazo	2,359	322,593	731.26	1,002	324,020	309.24	687	325,665	210.95	373.60
El Oro	3,578	485,385	737.15	1,321	490,136	269.52	716	495,269	144.57	328.45
Esmeraldas	764	350,731	217.83	342	356,012	96.06	357	361,615	98.72	135.39
Guayas	11,982	2,939,790	407.58	4,085	2,989,649	136.64	2,909	3,045,390	95.52	180.09

Imbabura	2,383	310,896	766.49	918	316,727	289.84	675	322,945	209.01	311.89
Loja	2,898	319,096	908.19	1,341	321,077	417.66	1,087	323,783	335.72	424.13
Los Ríos	1,978	596,520	331.59	832	602,672	138.05	546	611,232	89.33	171.80
Manabí	3,747	1,062,756	352.57	1,527	1,079,348	141.47	1,009	1,098,032	91.89	173.22
Morona Santiago	700	103,503	676.31	428	106,837	400.61	437	110,319	396.12	536.90
Napo	348	74,719	465.74	107	76,564	139.75	62	78,541	78.94	201.08
Pastaza	358	63,853	560.66	112	65,882	170.00	69	67,909	101.61	212.29
Pichincha	22,893	2,223,585	1029.55	7,544	2,251,613	335.05	5,154	2,284,974	225.56	403.89
Tungurahua	3,492	376,639	927.15	1,165	380,820	305.92	982	385,715	254.59	421.42
Zamora Chinchipe	312	66,892	466.42	203	68,498	296.36	143	70,194	203.72	283.17
Galápagos	93	19,815	469.34	22	20,163	109.11	25	20,552	121.64	205.14
Sucumbíos	621	121,567	510.83	201	122,721	163.79	137	123,687	110.76	239.73
Orellana	352	102,060	344.90	151	105,228	143.50	109	108,459	100.50	140.96
Santo Domingo de los Tsáchilas	1,967	310,768	632.95	828	316,233	261.83	494	322,081	153.38	295.24
Santa Elena	673	248,577	270.74	266	251,996	105.56	172	255,071	67.43	161.34
Total	71,108	11,336,115	627.27	26,749	11,492,486	232.75	19,270	11,669,852	165.13	283.14

Supplementary table S6. Acute respiratory infections in-hospital mortality rates by provinces and by calendar years, per 100,000 inhabitants for the adult sample.

**Provinces**	**2015**			**2016**			**2017**		
	**Cases**	**Population**	**Rates**	**Cases**	**Population**	**Rates**	**Cases**	**Population**	**Rates**
Azuay	129	484,441	26.63	166	495,771	33.48	183	506,740	36.11
Bolívar	12	123,092	9.75	15	124,667	12.03	13	126,084	10.31
Cañar	26	137,361	18.93	34	138,939	24.47	42	140,376	29.92
Carchi	28	110,792	25.27	23	113,451	20.27	14	116,146	12.05
Cotopaxi	33	261,830	12.60	25	268,886	9.30	23	275,933	8.34
Chimborazo	41	300,979	13.62	68	306,459	22.19	66	311,345	21.20
El Oro	122	426,747	28.59	120	438,960	27.34	132	451,069	29.26
Esmeraldas	22	313,627	7.01	26	320,526	8.11	28	327,269	8.56
Guayas	409	2,528,906	16.17	548	2,595,813	21.11	457	2,667,129	17.13
Imbabura	20	271,703	7.36	34	278,426	12.21	41	285,205	14.38
Loja	61	302,486	20.17	57	306,332	18.61	46	309,818	14.85
Los Ríos	47	518,868	9.06	39	533,171	7.31	42	547,951	7.66
Manabí	65	916,867	7.09	129	942,595	13.69	85	968,410	8.78
Morona Santiago	15	83,272	18.01	15	86,542	17.33	19	89,904	21.13
Napo	6	62,676	9.57	4	64,698	6.18	3	66,755	4.49
Pastaza	0	52,563	0.00	7	54,454	12.85	4	56,358	7.10
Pichincha	235	1,918,742	12.25	268	1,972,256	13.59	260	2,027,244	12.83
Tungurahua	104	342,949	30.33	76	349,462	21.75	79	355,746	22.21
Zamora Chinchipe	7	55,690	12.57	8	57,578	13.89	8	59,502	13.44
Galápagos	3	17,875	16.78	2	18,154	11.02	0	18,451	0.00
Sucumbíos	8	107,303	7.46	5	110,103	4.54	12	112,865	10.63
Orellana	4	83,703	4.78	1	86,691	1.15	3	89,687	3.34

Santo Domingo de los Tsáchilas	23	273,522	8.41	31	280,615	11.05	21	287,432	7.31
Santa Elena	17	213,260	7.97	27	220,134	12.27	16	226,953	7.05
Total	1,437	9,909,254	14.50	1,728	10,164,683	17.00	1,597	10,424,372	15.32

Supplementary table S6. (Continued**)**

Provinces	2018			2019			2020		
	Cases	Population	Rates	Cases	Population	Rates	Cases	Population	Rates
Azuay	162	518,930	31.22	148	530,686	27.89	945	536,960	175.99
Bolívar	8	127,316	6.28	13	128,404	10.12	86	129,214	66.56
Cañar	52	141,621	36.72	37	142,706	25.93	262	143,627	182.42
Carchi	25	118,510	21.10	26	120,222	21.63	156	121,005	128.92
Cotopaxi	41	282,864	14.49	29	289,718	10.01	164	296,248	55.36
Chimborazo	57	315,429	18.07	64	318,736	20.08	566	321,101	176.27
El Oro	86	462,595	18.59	78	472,876	16.49	1,175	480,375	244.60
Esmeraldas	20	333,727	5.99	25	339,856	7.36	274	345,531	79.30
Guayas	369	2,752,644	13.41	428	2,843,182	15.05	2,338	2,901,428	80.58
Imbabura	41	292,099	14.04	27	298,962	9.03	357	305,255	116.95
Loja	53	312,908	16.94	51	315,597	16.16	365	317,627	114.91
Los Ríos	50	565,055	8.85	25	582,101	4.29	298	591,876	50.35
Manabí	86	997,590	8.62	93	1,027,662	9.05	967	1,048,417	92.23
Morona Santiago	24	93,377	25.70	13	96,900	13.42	79	100,269	78.79
Napo	3	68,887	4.35	11	71,026	15.49	85	72,959	116.50
Pastaza	10	58,163	17.19	13	59,923	21.69	56	61,835	90.56

Pichincha	338	2,090,476	16.17	294	2,154,948	13.64	2,975	2,198,031	135.35
Tungurahua	109	362,053	30.11	91	368,116	24.72	921	372,929	246.96
Zamora Chinchipe	13	61,530	21.13	5	63,574	7.86	55	65,346	84.17
Galápagos	0	18,776	0.00	1	19,136	5.23	14	19,485	71.85
Sucumbíos	24	115,613	20.76	12	118,164	10.16	152	120,157	126.50
Orellana	2	92,732	2.16	5	95,842	5.22	59	98,951	59.63
Santo Domingo de los Tsáchilas	36	293,998	12.24	51	300,349	16.98	523	305,834	171.01
Santa Elena	24	233,372	10.28	11	239,279	4.60	211	244,366	86.35
Total	1,633	10,710,265	15.25	1,551	10,997,965	14.10	13,083	11,198,826	116.82

Supplementary table S6. (Continued**)**

**Provinces**	**2021**			**2022**			**2023**			**Average rates 2015-2023**
	**Cases**	**Population**	**Rates**	**Cases**	**Population**	**Rates**	**Cases**	**Population**	**Rates**	
Azuay	1,062	538,765	197.12	313	540,841	57.87	170	544,321	31.23	68.62
Bolívar	89	129,852	68.54	13	130,712	9.95	10	131,808	7.59	22.35
Cañar	205	144,353	142.01	51	145,202	35.12	35	146,285	23.93	57.72
Carchi	196	120,896	162.12	16	120,416	13.29	16	119,982	13.34	46.44
Cotopaxi	168	302,504	55.54	54	309,119	17.47	36	316,023	11.39	21.61
Chimborazo	503	322,593	155.92	103	324,020	31.79	42	325,665	12.90	52.45
El Oro	1,370	485,385	282.25	295	490,136	60.19	89	495,269	17.97	80.59
Esmeraldas	219	350,731	62.44	32	356,012	8.99	27	361,615	7.47	21.69
Guayas	1,910	2,939,790	64.97	551	2,989,649	18.43	338	3,045,390	11.10	28.66

Imbabura	442	310,896	142.17	72	316,727	22.73	29	322,945	8.98	38.65
Loja	638	319,096	199.94	143	321,077	44.54	70	323,783	21.62	51.97
Los Ríos	298	596,520	49.96	101	602,672	16.76	72	611,232	11.78	18.45
Manabí	1,094	1,062,756	102.94	284	1,079,348	26.31	125	1,098,032	11.38	31.12
Morona Santiago	67	103,503	64.73	29	106,837	27.14	14	110,319	12.69	30.99
Napo	70	74,719	93.68	13	76,564	16.98	6	78,541	7.64	30.54
Pastaza	81	63,853	126.85	10	65,882	15.18	4	67,909	5.89	33.04
Pichincha	3,370	2,223,585	151.56	724	2,251,613	32.15	280	2,284,974	12.25	44.42
Tungurahua	875	376,639	232.32	119	380,820	31.25	51	385,715	13.22	72.54
Zamora Chinchipe	67	66,892	100.16	20	68,498	29.20	15	70,194	21.37	33.76
Galápagos	16	19,815	80.75	1	20,163	4.96	3	20,552	14.60	22.80
Sucumbíos	177	121,567	145.60	37	122,721	30.15	18	123,687	14.55	41.15
Orellana	55	102,060	53.89	9	105,228	8.55	4	108,459	3.69	15.82
Santo Domingo de los Tsáchilas	592	310,768	190.50	153	316,233	48.38	72	322,081	22.35	54.25
Santa Elena	161	248,577	64.77	39	251,996	15.48	11	255,071	4.31	23.67
Total	13,725	11,336,115	121.07	3,182	11,492,486	27.69	1,537	11,669,852	13.17	39.44

Supplementary table S7. Acute respiratory infections hospitalizations rates by type of respiratory infection and by calendar years, per 100,000 inhabitants for the total study sample.

Type of respiratory infection	2015			2016			2017		
	Cases	Population	Rates	Cases	Population	Rates	Cases	Population	Rates
Acute upper respiratory infections	5,468	16,269,171	33.61	5,916	16,532,265	35.78	5,712	16,792,803	34.01
Influenza	106	16,269,171	0.65	487	16,532,265	2.95	713	16,792,803	4.25
Viral pneumonia	568	16,269,171	3.49	769	16,532,265	4.65	879	16,792,803	5.23
Bacterial pneumonia	6,830	16,269,171	41.98	8,322	16,532,265	50.34	8,807	16,792,803	52.45
Other Pneumonias due to other or unspecified organisms	27,692	16,269,171	170.21	32,195	16,532,265	194.74	30,200	16,792,803	179.84
Acute bronchitis	3,970	16,269,171	24.40	4,115	16,532,265	24.89	3,812	16,792,803	22.70
Acute bronchiolitis	1,919	16,269,171	11.80	2,394	16,532,265	14.48	2,437	16,792,803	14.51
Unspecified acute lower respiratory infection	1,247	16,269,171	7.66	1,678	16,532,265	10.15	2,042	16,792,803	12.16
Suppurative and necrotic conditions of lower respiratory tract	225	16,269,171	1.38	208	16,532,265	1.26	216	16,792,803	1.29
COVID-19	0	16,269,171	0.00	0	16,532,265	0.00	0	16,792,803	0.00
Total	48,025	16,269,171	295.19	56,084	16,532,265	339.24	54,818	16,792,803	326.44

Supplementary table S7. (Continued**)**

**Type of respiratory infection**	**2018**			**2019**			**2020**		
	**Cases**	**Population**	**Rates**	**Cases**	**Population**	**Rates**	**Cases**	**Population**	**Rates**
Acute upper respiratory infections	5,498	17,077,883	32.19	5,478	17,356,880	31.56	2,232	17,526,455	12.74
Influenza	2,242	17,077,883	13.13	1,654	17,356,880	9.53	519	17,526,455	2.96
Viral pneumonia	1,202	17,077,883	7.04	1,370	17,356,880	7.89	3,581	17,526,455	20.43
Bacterial pneumonia	11,104	17,077,883	65.02	10,738	17,356,880	61.87	4,812	17,526,455	27.46
Other Pneumonias due to other or unspecified organisms	31,524	17,077,883	184.59	29,264	17,356,880	168.60	15,206	17,526,455	86.76
Acute bronchitis	3,670	17,077,883	21.49	2,920	17,356,880	16.82	1,170	17,526,455	6.68
Acute bronchiolitis	2,271	17,077,883	13.30	2,488	17,356,880	14.33	875	17,526,455	4.99
Unspecified acute lower respiratory infection	2,497	17,077,883	14.62	2,737	17,356,880	15.77	1,322	17,526,455	7.54
Suppurative and necrotic conditions of lower respiratory tract	241	17,077,883	1.41	249	17,356,880	1.43	201	17,526,455	1.15
COVID-19	0	17,077,883	0.00	0	17,356,880	0.00	56,468	17,526,455	322.19
Total	60,249	17,077,883	352.79	56,898	17,356,880	327.81	86,386	17,526,455	492.89

Supplementary table S7. (Continued)

Type of respiratory infection	2021			2022			2023			Average rates 2015-2023
	Cases	Population	Rates	Cases	Population	Rates	Cases	Population	Rates	
Acute upper respiratory infections	1,659	17,614,396	9.42	5,515	17,715,301	31.13	5,622	17,834,831	31.52	28.00
Influenza	57	17,614,396	0.32	1,014	17,715,301	5.72	1,841	17,834,831	10.32	5.54
Viral pneumonia	1,867	17,614,396	10.60	2,693	17,715,301	15.20	3,941	17,834,831	22.10	10.74
Bacterial pneumonia	2,851	17,614,396	16.19	7,388	17,715,301	41.70	10,983	17,834,831	61.58	46.51
Other Pneumonias due to other or unspecified organisms	10,433	17,614,396	59.23	25,132	17,715,301	141.87	32,745	17,834,831	183.60	152.16
Acute bronchitis	1,019	17,614,396	5.79	2,730	17,715,301	15.41	2,530	17,834,831	14.19	16.93
Acute bronchiolitis	747	17,614,396	4.24	2,088	17,715,301	11.79	2,355	17,834,831	13.20	11.40
Unspecified acute lower respiratory infection	982	17,614,396	5.57	2,996	17,715,301	16.91	3,595	17,834,831	20.16	12.28
Suppurative and necrotic conditions of lower respiratory tract	209	17,614,396	1.19	317	17,715,301	1.79	341	17,834,831	1.91	1.42
COVID-19	63,615	17,614,396	361.15	14,153	17,715,301	79.89	2,317	17,834,831	12.99	194.06
Total	83,439	17,614,396	473.70	64,026	17,715,301	361.42	66,270	17,834,831	371.58	371.23

Supplementary table S8. Acute respiratory infections in-hospital mortality rates by type of respiratory infection and by calendar years, per 100,000 inhabitants for the total study sample.

**Type of respiratory infection**	**2015**			**2016**			**2017**		
	**Cases**	**Population**	**Rates**	**Cases**	**Population**	**Rates**	**Cases**	**Population**	**Rates**
Acute upper respiratory infections	4	16,269,171	0.02	11	16,532,265	0.07	12	16,792,803	0.07
Influenza	3	16,269,171	0.02	26	16,532,265	0.16	8	16,792,803	0.05
Viral pneumonia	10	16,269,171	0.06	12	16,532,265	0.07	5	16,792,803	0.03
Bacterial pneumonia	379	16,269,171	2.33	466	16,532,265	2.82	520	16,792,803	3.10
Other Pneumonias due to other or unspecified organisms	1,171	16,269,171	7.20	1,344	16,532,265	8.13	1,196	16,792,803	7.12
Acute bronchitis	9	16,269,171	0.06	10	16,532,265	0.06	10	16,792,803	0.06
Acute bronchiolitis	3	16,269,171	0.02	8	16,532,265	0.05	3	16,792,803	0.02
Unspecified acute lower respiratory infection	12	16,269,171	0.07	23	16,532,265	0.14	22	16,792,803	0.13
Suppurative and necrotic conditions of lower respiratory tract	12	16,269,171	0.07	12	16,532,265	0.07	16	16,792,803	0.10
COVID-19	0	16,269,171	0.00	0	16,532,265	0.00	0	16,792,803	0.00
Total	1,603	16,269,171	9.85	1,912	16,532,265	11.57	1,792	16,792,803	10.67

Supplementary table S8. (Continued)

Type of respiratory infection	2018			2019			2020		
	**Cases**	**Population**	**Rates**	**Cases**	**Population**	**Rates**	**Cases**	**Population**	**Rates**
Acute upper respiratory infections	3	17,077,883	0.02	8	17,356,880	0.05	13	17,526,455	0.07
Influenza	61	17,077,883	0.36	10	17,356,880	0.06	6	17,526,455	0.03
Viral pneumonia	18	17,077,883	0.11	27	17,356,880	0.16	630	17,526,455	3.59
Bacterial pneumonia	559	17,077,883	3.27	597	17,356,880	3.44	381	17,526,455	2.17
Other Pneumonias due to other or unspecified organisms	1,122	17,077,883	6.57	1,018	17,356,880	5.87	1,014	17,526,455	5.79
Acute bronchitis	6	17,077,883	0.04	1	17,356,880	0.01	9	17,526,455	0.05
Acute bronchiolitis	2	17,077,883	0.01	4	17,356,880	0.02	1	17,526,455	0.01
Unspecified acute lower respiratory infection	21	17,077,883	0.12	19	17,356,880	0.11	50	17,526,455	0.29
Suppurative and necrotic conditions of lower respiratory tract	16	17,077,883	0.09	18	17,356,880	0.10	12	17,526,455	0.07
COVID-19	0	17,077,883	0.00	0	17,356,880	0.00	11,145	17,526,455	63.59
Total	1,808	17,077,883	10.59	1,702	17,356,880	9.81	13,261	17,526,455	75.66

Supplementary table S8. (Continued)

Type of respiratory infection	2021			2022			2023			Average rates 2015-2023
	Cases	Population	Rates	Cases	Population	Rates	Cases	Population	Rates	
Acute upper respiratory infections	15	17,614,396	0.09	13	17,715,301	0.07	12	17,834,831	0.07	0.06
Influenza	4	17,614,396	0.02	7	17,715,301	0.04	9	17,834,831	0.05	0.09
Viral pneumonia	199	17,614,396	1.13	48	17,715,301	0.27	24	17,834,831	0.13	0.62
Bacterial pneumonia	371	17,614,396	2.11	446	17,715,301	2.52	450	17,834,831	2.52	2.70
Other Pneumonias due to other or unspecified organisms	732	17,614,396	4.16	1,039	17,715,301	5.86	1,050	17,834,831	5.89	6.29
Acute bronchitis	3	17,614,396	0.02	6	17,715,301	0.03	7	17,834,831	0.04	0.04
Acute bronchiolitis	12	17,614,396	0.07	2	17,715,301	0.01	0	17,834,831	0.00	0.02
Unspecified acute lower respiratory infection	18	17,614,396	0.10	25	17,715,301	0.14	27	17,834,831	0.15	0.14
Suppurative and necrotic conditions of lower respiratory tract	15	17,614,396	0.09	10	17,715,301	0.06	25	17,834,831	0.14	0.09
COVID-19	12,533	17,614,396	71.15	1,764	17,715,301	9.96	104	17,834,831	0.58	36.32
Total	13,902	17,614,396	78.92	3,360	17,715,301	18.97	1,708	17,834,831	9.58	26.18

Supplementary table S9. Acute respiratory infections hospitalizations rates by type of respiratory infection and by calendar years, per 100,000 inhabitants for the pediatric sample.

**Type of respiratory infection**	**2015**			**2016**			**2017**		
	**Cases**	**Population**	**Rates**	**Cases**	**Population**	**Rates**	**Cases**	**Population**	**Rates**
Acute upper respiratory infections	4,366	6,359,917	68.65	4,614	6,367,582	72.46	4,537	6,368,431	71.24
Influenza	42	6,359,917	0.66	173	6,367,582	2.72	532	6,368,431	8.35
Viral pneumonia	471	6,359,917	7.41	626	6,367,582	9.83	770	6,368,431	12.09
Bacterial pneumonia	3,932	6,359,917	61.82	4,668	6,367,582	73.31	4,684	6,368,431	73.55
Other Pneumonias due to other or unspecified organisms	16,903	6,359,917	265.77	20,447	6,367,582	321.11	18,691	6,368,431	293.49
Acute bronchitis	3,277	6,359,917	51.53	3,162	6,367,582	49.66	2,864	6,368,431	44.97
Acute bronchiolitis	1,873	6,359,917	29.45	2,332	6,367,582	36.62	2,379	6,368,431	37.36
Unspecified acute lower respiratory infection	1,064	6,359,917	16.73	1,436	6,367,582	22.55	1,788	6,368,431	28.08
Suppurative and necrotic conditions of lower respiratory tract	58	6,359,917	0.91	38	6,367,582	0.60	39	6,368,431	0.61
COVID-19	0	6,359,917	0.00	0	6,367,582	0.00	0	6,368,431	0.00
Total	31,986	6,359,917	502.93	37,496	6,367,582	588.86	36,284	6,368,431	569.75

Supplementary table S9. (Continued)

Type of respiratory infection	2018			2019			2020		
	Cases	Population	Rates	Cases	Population	Rates	Cases	Population	Rates
Acute upper respiratory infections	4,179	6,367,618	65.63	4,271	6,358,915	67.17	1,394	6,327,629	22.03
Influenza	1,111	6,367,618	17.45	939	6,358,915	14.77	242	6,327,629	3.82
Viral pneumonia	960	6,367,618	15.08	1,147	6,358,915	18.04	780	6,327,629	12.33
Bacterial pneumonia	6,050	6,367,618	95.01	5,662	6,358,915	89.04	2,409	6,327,629	38.07
Other Pneumonias due to other or unspecified organisms	18,923	6,367,618	297.18	18,206	6,358,915	286.31	8,037	6,327,629	127.01
Acute bronchitis	2,596	6,367,618	40.77	2,078	6,358,915	32.68	748	6,327,629	11.82
Acute bronchiolitis	2,212	6,367,618	34.74	2,439	6,358,915	38.36	858	6,327,629	13.56
Unspecified acute lower respiratory infection	2,083	6,367,618	32.71	2,265	6,358,915	35.62	671	6,327,629	10.60
Suppurative and necrotic conditions of lower respiratory tract	65	6,367,618	1.02	67	6,358,915	1.05	28	6,327,629	0.44
COVID-19	0	6,367,618	0.00	0	6,358,915	0.00	2,431	6,327,629	38.42
Total	38,179	6,367,618	599.58	37,074	6,358,915	583.02	17,598	6,327,629	278.11

Supplementary table S9. (Continued)

Type of respiratory infection	2021			2022			2023			Average rates 2015-2023
	Cases	Population	Rates	Cases	Population	Rates	Cases	Population	Rates	
Acute upper respiratory infections	1,176	6,278,281	18.73	4,592	6,222,815	73.79	4,597	6,164,979	74.57	59.36
Influenza	30	6,278,281	0.48	749	6,222,815	12.04	1,319	6,164,979	21.40	9.08
Viral pneumonia	727	6,278,281	11.58	2,254	6,222,815	36.22	3,564	6,164,979	57.81	20.04
Bacterial pneumonia	880	6,278,281	14.02	4,251	6,222,815	68.31	7,255	6,164,979	117.68	70.09
Other Pneumonias due to other or unspecified organisms	5,291	6,278,281	84.27	16,166	6,222,815	259.79	22,107	6,164,979	358.59	254.84
Acute bronchitis	804	6,278,281	12.81	2,223	6,222,815	35.72	2,076	6,164,979	33.67	34.85
Acute bronchiolitis	739	6,278,281	11.77	2,054	6,222,815	33.01	2,330	6,164,979	37.79	30.30
Unspecified acute lower respiratory infection	818	6,278,281	13.03	2,612	6,222,815	41.97	3,109	6,164,979	50.43	27.97
Suppurative and necrotic conditions of lower respiratory tract	29	6,278,281	0.46	69	6,222,815	1.11	93	6,164,979	1.51	0.86
COVID-19	1,837	6,278,281	29.26	2,307	6,222,815	37.07	550	6,164,979	8.92	28.42
Total	12,331	6,278,281	196.41	37,277	6,222,815	599.04	47,000	6,164,979	762.37	520.01

Supplementary table S10. Acute respiratory infections in-hospital mortality rates by type of respiratory infection and by calendar years, per 100,000 inhabitants for the pediatric sample.

**Type of respiratory infection**	**2015**			**2016**			**2017**		
	**Cases**	**Population**	**Rates**	**Cases**	**Population**	**Rates**	**Cases**	**Population**	**Rates**
Acute upper respiratory infections	2	6,359,917	0.03	4	6,367,582	0.06	6	6,368,431	0.09
Influenza	1	6,359,917	0.02	4	6,367,582	0.06	3	6,368,431	0.05
Viral pneumonia	3	6,359,917	0.05	2	6,367,582	0.03	1	6,368,431	0.02
Bacterial pneumonia	34	6,359,917	0.53	26	6,367,582	0.41	33	6,368,431	0.52
Other Pneumonias due to other or unspecified organisms	108	6,359,917	1.70	131	6,367,582	2.06	130	6,368,431	2.04
Acute bronchitis	5	6,359,917	0.08	2	6,367,582	0.03	2	6,368,431	0.03
Acute bronchiolitis	3	6,359,917	0.05	3	6,367,582	0.05	2	6,368,431	0.03
Unspecified acute lower respiratory infection	8	6,359,917	0.13	11	6,367,582	0.17	17	6,368,431	0.27
Suppurative and necrotic conditions of lower respiratory tract	2	6,359,917	0.03	1	6,367,582	0.02	1	6,368,431	0.02
COVID-19	0	6,359,917	0.00	0	6,367,582	0.00	0	6,368,431	0.00
Total	166	6,359,917	2.61	184	6,367,582	2.89	195	6,368,431	3.06

Supplementary table S10. (Continued)

Type of respiratory infection	2018			2019			2020		
	**Cases**	**Population**	**Rates**	**Cases**	**Population**	**Rates**	**Cases**	**Population**	**Rates**
Acute upper respiratory infections	0	6,367,618	0.00	3	6,358,915	0.05	2	6,327,629	0.03
Influenza	7	6,367,618	0.11	2	6,358,915	0.03	1	6,327,629	0.02
Viral pneumonia	6	6,367,618	0.09	3	6,358,915	0.05	9	6,327,629	0.14
Bacterial pneumonia	37	6,367,618	0.58	44	6,358,915	0.69	29	6,327,629	0.46
Other Pneumonias due to other or unspecified organisms	111	6,367,618	1.74	95	6,358,915	1.49	79	6,327,629	1.25
Acute bronchitis	1	6,367,618	0.02	0	6,358,915	0.00	0	6,327,629	0.00
Acute bronchiolitis	2	6,367,618	0.03	3	6,358,915	0.05	0	6,327,629	0.00
Unspecified acute lower respiratory infection	8	6,367,618	0.13	1	6,358,915	0.02	1	6,327,629	0.02
Suppurative and necrotic conditions of lower respiratory tract	3	6,367,618	0.05	0	6,358,915	0.00	0	6,327,629	0.00
COVID-19	0	6,367,618	0.00	0	6,358,915	0.00	57	6,327,629	0.90
Total	175	6,367,618	2.75	151	6,358,915	2.37	178	6,327,629	2.81

Supplementary table S10. (Continued)

Type of respiratory infection	2021			2022			2023			Average rates 2015-2023
	Cases	Population	Rates	Cases	Population	Rates	Cases	Population	Rates	
Acute upper respiratory infections	8	6,278,281	0.13	4	6,222,815	0.06	4	6,164,979	0.06	0.06
Influenza	0	6,278,281	0.00	1	6,222,815	0.02	3	6,164,979	0.05	0.04
Viral pneumonia	3	6,278,281	0.05	10	6,222,815	0.16	4	6,164,979	0.06	0.07
Bacterial pneumonia	30	6,278,281	0.48	40	6,222,815	0.64	46	6,164,979	0.75	0.56
Other Pneumonias due to other or unspecified organisms	69	6,278,281	1.10	94	6,222,815	1.51	102	6,164,979	1.65	1.62
Acute bronchitis	2	6,278,281	0.03	3	6,222,815	0.05	5	6,164,979	0.08	0.04
Acute bronchiolitis	12	6,278,281	0.19	2	6,222,815	0.03	0	6,164,979	0.00	0.05
Unspecified acute lower respiratory infection	4	6,278,281	0.06	1	6,222,815	0.02	2	6,164,979	0.03	0.09
Suppurative and necrotic conditions of lower respiratory tract	1	6,278,281	0.02	1	6,222,815	0.02	2	6,164,979	0.03	0.02
COVID-19	48	6,278,281	0.76	22	6,222,815	0.35	3	6,164,979	0.05	0.52
Total	177	6,278,281	2.82	178	6,222,815	2.86	171	6,164,979	2.77	2.77

Supplementary table S11. Acute respiratory infections hospitalizations rates by type of respiratory infection and by calendar years, per 100,000 inhabitants for the adult sample.

**Type of respiratory infection**	**2015**			**2016**			**2017**		
	**Cases**	**Population**	**Rates**	**Cases**	**Population**	**Rates**	**Cases**	**Population**	**Rates**
Acute upper respiratory infections	1,102	9,909,254	11.12	1,302	10,164,683	12.81	1,175	10,424,372	11.27
Influenza	64	9,909,254	0.65	314	10,164,683	3.09	181	10,424,372	1.74
Viral pneumonia	97	9,909,254	0.98	143	10,164,683	1.41	109	10,424,372	1.05
Bacterial pneumonia	2,898	9,909,254	29.25	3,654	10,164,683	35.95	4,123	10,424,372	39.55
Other Pneumonias due to other or unspecified organisms	10,789	9,909,254	108.88	11,748	10,164,683	115.58	11,509	10,424,372	110.40
Acute bronchitis	693	9,909,254	6.99	953	10,164,683	9.38	948	10,424,372	9.09
Acute bronchiolitis	46	9,909,254	0.46	62	10,164,683	0.61	58	10,424,372	0.56
Unspecified acute lower respiratory infection	183	9,909,254	1.85	242	10,164,683	2.38	254	10,424,372	2.44
Suppurative and necrotic conditions of lower respiratory tract	167	9,909,254	1.69	170	10,164,683	1.67	177	10,424,372	1.70
COVID-19	0	9,909,254	0.00	0	10,164,683	0.00	0	10,424,372	0.00
Total	16,039	9,909,254	161.86	18,588	10,164,683	182.87	18,534	10,424,372	177.79

Supplementary table S11. (Continued)

Type of respiratory infection	2018			2019			2020		
	**Cases**	**Population**	**Rates**	**Cases**	**Population**	**Rates**	**Cases**	**Population**	**Rates**
Acute upper respiratory infections	1,319	10,710,265	12.32	1,207	10,997,965	10.97	838	11,198,826	7.48
Influenza	1,131	10,710,265	10.56	715	10,997,965	6.50	277	11,198,826	2.47
Viral pneumonia	242	10,710,265	2.26	223	10,997,965	2.03	2,801	11,198,826	25.01
Bacterial pneumonia	5,054	10,710,265	47.19	5,076	10,997,965	46.15	2,403	11,198,826	21.46
Other Pneumonias due to other or unspecified organisms	12,601	10,710,265	117.65	11,058	10,997,965	100.55	7,169	11,198,826	64.02
Acute bronchitis	1,074	10,710,265	10.03	842	10,997,965	7.66	422	11,198,826	3.77
Acute bronchiolitis	59	10,710,265	0.55	49	10,997,965	0.45	17	11,198,826	0.15
Unspecified acute lower respiratory infection	414	10,710,265	3.87	472	10,997,965	4.29	651	11,198,826	5.81
Suppurative and necrotic conditions of lower respiratory tract	176	10,710,265	1.64	182	10,997,965	1.65	173	11,198,826	1.54
COVID-19	0	10,710,265	0.00	0	10,997,965	0.00	54,037	11,198,826	482.52
Total	22,070	10,710,265	206.06	19,824	10,997,965	180.25	68,788	11,198,826	614.24

Supplementary table S11. (Continued)

Type of respiratory infection	2021			2022			2023			Average rates 2015-2023
	Cases	Population	Rates	Cases	Population	Rates	Cases	Population	Rates	
Acute upper respiratory infections	483	11,336,115	4.26	923	11,492,486	8.03	1,025	11,669,852	8.78	9.67
Influenza	27	11,336,115	0.24	265	11,492,486	2.31	522	11,669,852	4.47	3.56
Viral pneumonia	1,140	11,336,115	10.06	439	11,492,486	3.82	377	11,669,852	3.23	5.54
Bacterial pneumonia	1,971	11,336,115	17.39	3,137	11,492,486	27.30	3,728	11,669,852	31.95	32.91
Other Pneumonias due to other or unspecified organisms	5,142	11,336,115	45.36	8,966	11,492,486	78.02	10,638	11,669,852	91.16	92.40
Acute bronchitis	215	11,336,115	1.90	507	11,492,486	4.41	454	11,669,852	3.89	6.35
Acute bronchiolitis	8	11,336,115	0.07	34	11,492,486	0.30	25	11,669,852	0.21	0.37
Unspecified acute lower respiratory infection	164	11,336,115	1.45	384	11,492,486	3.34	486	11,669,852	4.16	3.29
Suppurative and necrotic conditions of lower respiratory tract	180	11,336,115	1.59	248	11,492,486	2.16	248	11,669,852	2.13	1.75
COVID-19	61,778	11,336,115	544.97	11,846	11,492,486	103.08	1,767	11,669,852	15.14	286.43
Total	71,108	11,336,115	627.27	26,749	11,492,486	232.75	19,270	11,669,852	165.13	283.14

Supplementary table S12. Acute respiratory infections in-hospital mortality rates by type of respiratory infection and by calendar years, per 100,000 inhabitants for the adult sample.

**Type of respiratory infection**	**2015**			**2016**			**2017**		
	**Cases**	**Population**	**Rates**	**Cases**	**Population**	**Rates**	**Cases**	**Population**	**Rates**
Acute upper respiratory infections	2	9,909,254	0.02	7	10,164,683	0.07	6	10,424,372	0.06
Influenza	2	9,909,254	0.02	22	10,164,683	0.22	5	10,424,372	0.05
Viral pneumonia	7	9,909,254	0.07	10	10,164,683	0.10	4	10,424,372	0.04
Bacterial pneumonia	345	9,909,254	3.48	440	10,164,683	4.33	487	10,424,372	4.67
Other Pneumonias due to other or unspecified organisms	1,063	9,909,254	10.73	1,213	10,164,683	11.93	1,066	10,424,372	10.23
Acute bronchitis	4	9,909,254	0.04	8	10,164,683	0.08	8	10,424,372	0.08
Acute bronchiolitis	0	9,909,254	0.00	5	10,164,683	0.05	1	10,424,372	0.01
Unspecified acute lower respiratory infection	4	9,909,254	0.04	12	10,164,683	0.12	5	10,424,372	0.05
Suppurative and necrotic conditions of lower respiratory tract	10	9,909,254	0.10	11	10,164,683	0.11	15	10,424,372	0.14
COVID-19	0	9,909,254	0.00	0	10,164,683	0.00	0	10,424,372	0.00
Total	1,437	9,909,254	14.50	1,728	10,164,683	17.00	1,597	10,424,372	15.32

Supplementary table S12. (Continued**)**

Type of respiratory infection	2018			2019			2020		
	Cases	Population	Rates	Cases	Population	Rates	Cases	Population	Rates
Acute upper respiratory infections	3	10,710,265	0.03	5	10,997,965	0.05	11	11,198,826	0.10
Influenza	54	10,710,265	0.50	8	10,997,965	0.07	5	11,198,826	0.04
Viral pneumonia	12	10,710,265	0.11	24	10,997,965	0.22	621	11,198,826	5.55
Bacterial pneumonia	522	10,710,265	4.87	553	10,997,965	5.03	352	11,198,826	3.14
Other Pneumonias due to other or unspecified organisms	1,011	10,710,265	9.44	923	10,997,965	8.39	935	11,198,826	8.35
Acute bronchitis	5	10,710,265	0.05	1	10,997,965	0.01	9	11,198,826	0.08
Acute bronchiolitis	0	10,710,265	0.00	1	10,997,965	0.01	1	11,198,826	0.01
Unspecified acute lower respiratory infection	13	10,710,265	0.12	18	10,997,965	0.16	49	11,198,826	0.44
Suppurative and necrotic conditions of lower respiratory tract	13	10,710,265	0.12	18	10,997,965	0.16	12	11,198,826	0.11
COVID-19	0	10,710,265	0.00	0	10,997,965	0.00	11,088	11,198,826	99.01
Total	1,633	10,710,265	15.25	1,551	10,997,965	14.10	13,083	11,198,826	116.82

Supplementary table S12. (Continued)

Type of respiratory infection	2021			2022			2023			Average rates 2015-2023
	Cases	Population	Rates	Cases	Population	Rates	Cases	Population	Rates	
Acute upper respiratory infections	7	11,336,115	0.06	9	11,492,486	0.08	8	11,669,852	0.07	0.06
Influenza	4	11,336,115	0.04	6	11,492,486	0.05	6	11,669,852	0.05	0.12
Viral pneumonia	196	11,336,115	1.73	38	11,492,486	0.33	20	11,669,852	0.17	0.92
Bacterial pneumonia	341	11,336,115	3.01	406	11,492,486	3.53	404	11,669,852	3.46	3.95
Other Pneumonias due to other or unspecified organisms	663	11,336,115	5.85	945	11,492,486	8.22	948	11,669,852	8.12	9.03
Acute bronchitis	1	11,336,115	0.01	3	11,492,486	0.03	2	11,669,852	0.02	0.04
Acute bronchiolitis	0	11,336,115	0.00	0	11,492,486	0.00	0	11,669,852	0.00	0.01
Unspecified acute lower respiratory infection	14	11,336,115	0.12	24	11,492,486	0.21	25	11,669,852	0.21	0.16
Suppurative and necrotic conditions of lower respiratory tract	14	11,336,115	0.12	9	11,492,486	0.08	23	11,669,852	0.20	0.13
COVID-19	12,485	11,336,115	110.13	1,742	11,492,486	15.16	101	11,669,852	0.87	56.29
Total	13,725	11,336,115	121.07	3,182	11,492,486	27.69	1,537	11,669,852	13.17	39.44
